# Fabrication of Co_3_O_4_/P-rGO for electrocatalytic reduction of greenhouse CO_2_ gas into value-added chemicals in aqueous solution

**DOI:** 10.1039/d6ra01555g

**Published:** 2026-04-13

**Authors:** Rad Mosharrof Mim, Md. Shamim Alam, Sangjukta Yesmin, Md. Mominul Islam, Chanchal Kumar Roy, Abu Bin Imran, Al-Nakib Chowdhury

**Affiliations:** a Department of Chemistry, Bangladesh University of Engineering and Technology Dhaka-1000 Bangladesh abimran@chem.buet.ac.bd nakib@chem.buet.ac.bd; b Department of Textile Engineering, Southeast University Tejgaon I/A Dhaka Bangladesh; c Department of Chemistry, University of Dhaka Dhaka-1000 Bangladesh

## Abstract

The electrocatalytic reduction of greenhouse CO_2_ gas into value-added fuels or chemical feedstocks sustainably addresses energy and environmental crises. However, CO_2_ reduction is particularly effective with electrocatalysts, which exhibit distinct functionality at electrode surfaces. In this work, we demonstrate the electrocatalytic reduction of CO_2_ using flower-like cobalt oxide (Co_3_O_4_) synthesized *via* a hydrothermal method. Co_3_O_4_ is incorporated into P-doped rGO *via* ultrasonication to form a hybrid electrocatalyst, thereby enhancing CO_2_ reduction efficiency by improving electrode surface functionality. Chemical, morphological, and structural characterization of the synthesized catalyst was carried out using scanning electron microscopy (SEM), X-ray diffraction (XRD), Raman spectroscopy, and X-ray photoelectron spectroscopy (XPS) analysis. The electrocatalytic reduction of CO_2_ was performed in 0.5 M NaHCO_3_ aqueous solution at a pH of 7.5 (CO_2_ conditions) in a three-electrode system and applying potential *vs.* Ag/AgCl (3 M KCl sat.) as the reference electrode, platinum wire as the counter electrode, and the prepared catalyst as a modified graphite working electrode. Chronoamperometry shows CO_2_ conversion stability under a constant voltage of −0.62 V (*vs.* Ag/AgCl) for 2.5 hours. The Co_3_O_4_ catalyst primarily yields ethanoic acid with 69% faradaic efficiency at a current density of −0.5 mA cm^−2^. Additionally, ethanoic acid and propanal are detected for the hybrid flower-like Co_3_O_4_/P-rGO catalyst, with 58% and 9% faradaic efficiencies at a constant current density of −0.8 mA cm^−2^. These results highlight that incorporating Co_3_O_4_ into the P-rGO improves reduction performance. This can provide a promising platform for synthesizing and fabricating shape-based materials as an electrocatalyst, paving the way for a future powered by renewable, boundless energy and wealth from greenhouse CO_2_ gas and other pollutants.

## Abstract 2

The electrocatalytic reduction of greenhouse CO_2_ gas into value-added fuels.

## Graphical abstract

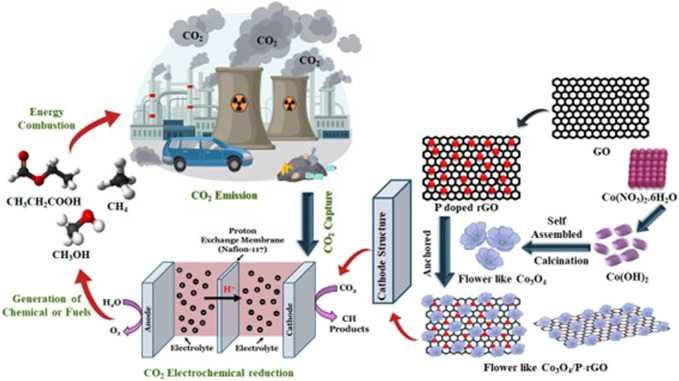

## 1. Introduction

The increasing atmospheric CO_2_ is a harmful greenhouse gas and the main contributor to global warming.^1^ Global warming leads to a series of problems, such as environmental pollution, ozone layer depletion, extreme weather, and desertification.^2^ Conversely, nature maintains a delicate balance in the carbon cycle by regulating the consumption and emission of CO_2_ from various sources. Anthropogenic activities, particularly fossil fuel combustion and industrial processes, emit approximately 37.4 Gt of CO_2_ per year, accounting for about 65% of total greenhouse gas emissions, and this cannot be accommodated in the natural carbon cycle.^3^ To keep humanity safe and secure, mitigating the challenge of increasing CO_2_ is now becoming a global concern. In managing this, converting CO_2_ into value-added chemicals or fuels appears to be the most attractive to scientists and technologists.^4^

In this regard, the conversion of captured CO_2_*via* chemical, photochemical, electrochemical, and biochemical processes appears to be extremely prevalent in modern technology.^5^ Compared to other conversion techniques such as thermal and photochemical processes, electrocatalytic reduction of CO_2_ is a simple, eco-friendly, and cost-effective approach that provides high-value reduction products with high conversion stability.^6,7^ A much higher negative potential of −1.9 V is needed for the electrochemical reduction of CO_2_.^8^ This potential may increase due to the overpotential and kinetic barrier of the electrode used in the electrochemical approach for CO_2_ reduction. To resolve these problems, a strong electrocatalyst is highly needed, combining high faradaic efficiency and good conversion stability with reduced reaction overpotential for the electrochemical reduction of CO_2_. Several catalysts, such as bimetallic compounds^9,10^ metal oxide derivatives^10,11^ and metal–organic hybrid materials^12,13^ have already been used for the electrocatalytic reduction of CO_2_. Among these, metal oxide-based catalysts are very efficient due to their high product selectivity, high conversion efficiencies, and low overpotential for the reduction of CO_2_ compared to pure metallic forms. Various metal oxide-based catalysts, such as CuO,^14^ Cu_2_O,^15^ Sn_3_O_4_,^16^ SnO_2_,^17^ NiO,^18^ Pb_3_O_4_,^19^ Co_3_O_4_,^20^ MnO_2_,^21^ Fe_3_O_4_ (ref. 22) are used for the electrochemical reduction of CO_2_. Due to low cost, most abundant and high catalytic activity, a shape-controlled Co_3_O_4_ is promising for CO_2_ conversion yielding methanol, formic acid, and ethanol as the value-added chemicals.^23^ Co_3_O_4_ is well suited for heterogeneous catalysis because of its redox reactivity. The dual oxidation state^24^ Co^2+^/Co^3+^ can provide thermodynamic stability to the Co_3_O_4_ catalyst.^25,26^ With that, the d orbitals of Co ions grant more active sites for conducting various reactions to function as an oxidizing or reducing character at ambient conditions.^24^ Various morphology-based Co_3_O_4,_ such as nanofibrous,^27^ crystal facet-tailored,^28^ self-templated hierarchical nanosheets,^29^ hollow multi-shelled structured,^30^ nano-cube^31^ like Co_3_O_4_ have already been used as an electrocatalyst for the electrochemical reduction of CO_2_. It is quite certain that Co_3_O_4_ can play a strong role as an electrocatalyst, but the Co ion suffers agglomeration problems in electrolyte solutions, and it creates low conductivity, which can reduce the transportation of electrons and its active sites during the reaction time.^32,33^

Thus, if a support of another conductive material, such as graphene oxide (GO),^34^ reduced graphene oxide (rGO),^35^ poly-aniline,^36^ g-C_3_N_4_,^37^ is incorporated into a metal substrate, then the reduction might be more effective. Among the supports, rGO is the most attractive in CO_2_ conversion due to its high surface area, intrinsic mobility, excellent mechanical strength, and thermal conductivity.^38^ The large surface area of rGO enhances CO_2_ adsorption on the electrode surface. The adsorption of CO_2_ on the electrode surface can tune the conversion efficiencies or the faradaic efficiencies of selective products.^39^ On the other hand, doping rGO with foreign materials like nitrogen (N), phosphorus (P), and sulphur (S) can modulate the electronic aspects of the rGO and thus make it more conductive than its undoped state. Heteroatoms with different electronegativity N (3), S (2.5), P (2.1) can break the electroneutrality of graphene derivatives, and show a tremendous role as a supporting material for the different electrochemical applications, including water splitting, batteries, supercapacitors, and fuel cells.^40,41^ P is an efficient dopant for its electronic stability with changeable electronic valences. It also has lower electronegativity from carbon and high electron-donating ability, which can modulate the carbon atom's electronic surrounding and local charge density in rGO materials.^42^ Incorporation of P into graphene derivatives can induce more active sites due to the larger size and lower electronegativity of P than any other dopant. Not only the active sites, but also the lone pair of P can provide a conjugation with the graphene π system, and that can also modulate the band structure, electrical and chemical reactivity of graphene derivatives.^43,44^ The conjugation between dopant and substrate might be particularly worthy cause the impact of any heteroatom doping of graphene significantly depends on the nature of bonding involved. G. Bharath *et al.* have shown that using self-assembled Co_3_O_4_ nanospheres on N-doped reduced graphene oxide (Co_3_O_4_/N-rGO) bifunctional catalyst has shown an excellent application for CO_2_ reduction, yielding methanol with 74.8% faradaic efficiency.^45^ Hence, it is expected that, when rGO support is doped with P and incorporated into the flower-like Co_3_O_4_, it would be able to yield a potential hybrid catalyst. In that case, the hybrid should perform more than the single entity present in the hybrid catalyst.

This study introduces a novel approach of morphology-based work for the electrochemical reduction of CO_2_, which has not previously been reported. In this regard, the flower-like Co_3_O_4_ and flower-like Co_3_O_4_/P-rGO hybrid catalyst will be synthesized using hydrothermal and ultrasonication methods, respectively, and evaluated as a cathode for the electrochemical reduction of greenhouse CO_2_ gas. Various analytical methods, including XRD, FESEM, EDX, Raman, and XPS, are used to assess the chemical and morphological aspects of the catalyst. Therefore, catalytic stability, product selectivity, and faradaic efficiency of produced value-added chemicals or fuels will be analyzed to comprehensively evaluate the performance of the electrocatalytic reduction of CO_2_ gas, providing insights into their potential for sustainable CO_2_ conversion to useful chemicals and fuels.

## 2. Materials and methods

### 2.1 Materials

Sodium nitrate (NaNO_3_), hexamethylenetetramine (C_6_H_12_N_4_), cobalt nitrate hexahydrate (Co(NO_3_)_2_·6H_2_O), absolute ethanol (C_2_H_5_OH) (G.R. 99.7%), graphite powder, nitric acid (HNO_3_), sulfuric acid (H_2_SO_4_), potassium permanganate (KMnO_4_), hydrogen peroxide (H_2_O_2_), di-potassium hydrogen phosphate (K_2_HPO_4_), deionized water, sodium bicarbonate (NaHCO_3_), Nafion-117, dimethylsulfoxide (DMSO, A.R., 99.5%) were purchased from Sigma-Aldrich, India. All chemicals were used without further purification. The CO_2_ and N_2_ (purity: 99.99%) gases were purchased from Essence Industrial Gas Limited, Dhaka, Bangladesh.

### 2.2 Synthesis of Co_3_O_4_

A facile low-temperature hydrothermal process is used to synthesize flower-like Co_3_O_4_. The molar ratio of precursors (NaNO_3_, HMTA, Co(NO_3_)_2_·6H_2_O) was maintained at 1 : 1 : 0.2. 0.85 g of NaNO_3_ and 1.7776 g of HMTA were dissolved in a mixture of 40 mL of deionized water and 5 mL of anhydrous ethanol, followed by the addition of 0.58 g of Co (NO_3_)_2_·6H_2_O. The obtained light pink colored homogeneous solution was then transferred into a 100 mL Teflon-lined stainless autoclave for hydrothermal treatment at 110 °C for 3 h. Subsequently, the autoclave cooled to ambient temperature, and the resultant green precipitate was collected by centrifugation and washed three times with water and anhydrous ethanol. The green residue was dried at 70 °C for 10 h. Finally, the dried green powder of cobalt was calcined at 400 °C for 2 h in ambient atmosphere, and the black Co_3_O_4_ samples were obtained.

### 2.3 Synthesis of P-doped rGO

GO is synthesized by harsh oxidation of the graphite powders by modified Hummers' method.^46^ Then, the P-doped rGO is synthesized by a conventional one-step hydrothermal method. 200 mg GO was dispersed in 60 mL deionized water by sonicating in an ultrasonic bath at 10 kHz frequency until a homogeneous mixture was obtained. To this dispersion, 1 g, 2 g, and 4 g of di-potassium hydrogen phosphate were added separately to the GO suspension and further sonicated for 2 h. Each resulting suspension was transferred to the 100 mL Teflon-lined stainless autoclave for hydrothermal treatment at 180 °C for 12 h. Subsequently, the autoclave cooled to ambient temperature, and the resultant black precipitate was filtered and washed with deionized water and ethanol several times. Finally, the resultant black precipitates of P-doped rGO (P-rGO) were dried at 60 °C for 12 h in a vacuum oven. Here, the obtained samples were labeled as P_(0.5%)_rGO, P_(1%)_rGO, and P_(2%)_rGO corresponding to the increase of P content.

### 2.4 Synthesis of shape controlled Co_3_O_4_/P doped rGO

30 mg of P-rGO with varying P content P_(0.5%)_rGO, P_(1%)_rGO and P_(2%)_rGO was mixed separately to 90 mg of Co_3_O_4_ in 120 mL ethanol. The resultant mixture was ultrasonicated at 10 kHz frequency for 5 h. Initially, the suspension was sonicated for 2.5 h at room temperature and then at 60 °C. The suspension was then dried at 70 °C in a vacuum oven. The powder was washed with deionized water three times and further dried at 70 °C for 12 h in a vacuum oven. As a result, Co_3_O_4_/P-rGO hybrid composite with different phosphorus contents was successfully obtained and designated as Co_3_O_4_/P_(0.5%)_rGO, Co_3_O_4_/P_(1%)_rGO, and Co_3_O_4_/P_(2%)_rGO, respectively.

### 2.5 Characterizations

The phase purity levels and crystal structures of the synthesized flower-like Co_3_O_4_, P-rGO and flower-like Co_3_O_4_/P-rGO materials were investigated using an X-ray diffractometer (XRD, Rigaku Ultima IV, Japan) with Cu–K-alpha radiation and operating at a constant current of 40 mA and a constant voltage of 40 kV. The morphological aspect of the synthesized samples was investigated by a field emission scanning electron microscope (FESEM, ZEISS Gemini SEM, Germany) coupled with an Xmass detector for energy-dispersive X-ray spectroscopy (EDX) analysis to determine the elemental composition of the prepared samples. The Raman spectra were recorded using a micro-Raman spectrometer (Renishaw InVia Reflex 532) with an incident wavelength of 532.5 nm. The laser power was set to 0.1 mW to avoid potential damage or unexpected reductions due to laser irradiation. The graphene samples were placed on a background-free glass slide. The accumulation time of each spectrum was 100 s. To determine the chemical states of the elements and the electronic structure of the prepared composite. Thermo Scientific ESCALAB 250Xi XPS Microprobe, integrated with an Al Kα micro focusing X-ray monochromator, was used.

### 2.6 Electrode preparation

**Fig. 1:**
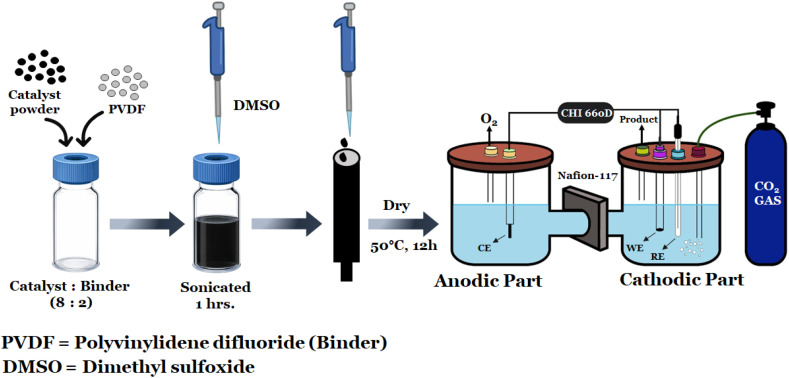
Fabrication of the electrode.

The electrode was prepared by the drop-casting method. Briefly, catalyst powder was used as an active material, and a binder and a solvent were used to fabricate the electrode. The binder's purpose was to ensure the active material remained on the electrode in the electrolyte solution. The working electrodes were prepared using a synthesized catalyst and binder with a wt% ratio of approximately 80 : 20. The polyvinylidene fluoride (PVDF) and DMSO were used as binder and solvent. To prepare the flower-like Co_3_O_4_ and Co_3_O_4_/P-rGO catalyst ink, 0.01 g of catalyst powder, 0.0024 g PVDF, and 160 µL DMSO were mixed and subjected to ultrasonic treatment at a power of 10 kHz for 30 min at room temperature (25 °C) to ensure uniform homogeneity. Subsequently, a calculated volume of the catalyst ink (*e.g.*, 20–40 µL) was drop-cast onto a pre-polished graphite disk electrode in an area of 0.52 cm^2^ to achieve a catalyst loading of 0.5 mg cm^−2^. The electrodes were dried in a vacuum oven at 50 °C for 12 h. Now, the dried electrodes were ready for electrochemical characterization, as shown in schematic Fig. 1.

### 2.7 Electrochemical performance analysis

**Fig. 2:**
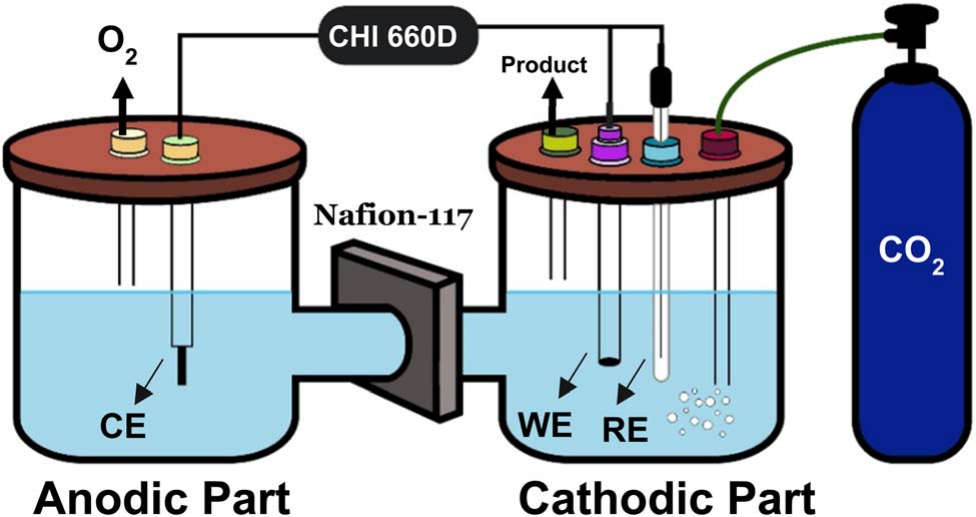
Schematic diagram of the electrochemical setup for CO_2_ reduction.

To evaluate the electrochemical response for CO_2_ reduction, a standard three-electrode setup was employed in an electrochemical H-type cell where the prepared electrode served as the working electrode (WE), Pt wire as a counter electrode (CE), and Ag/AgCl (3 M KCl) as the reference electrode (RE). The two compartments (anodic and cathodic) of the electrochemical H-type cell were separated by a proton exchange membrane (Nafion-117) to control the back oxidation of the counter electrode in the anodic compartment. All measurements were performed in a 0.5 M NaHCO3 aqueous solution, which served as the electrolyte. The electrocatalytic performance of the prepared electrode was measured using a CHI-660D electrochemical workstation. To investigate the electrocatalytic characteristics of CO_2_ reduction, the reduced current density of the catalyst in a CO_2_ medium was initially measured by using linear sweep voltammetry techniques. Linear sweep voltammetry was conducted in a 0.5 M NaHCO_3_ solution, which was saturated with N_2_ and CO_2_ by bubbling for 2 h before the experiment. Linear sweep voltammetry measurements were implemented in a conventional three-electrode cell where a prepared electrode was used as the WE, Ag/AgCl (3 M KCl) as the RE, and Pt wire as the CE with a scan rate of 30 mV s^−1^. To evaluate the electrocatalytic reduction of CO_2_, chronoamperometry was performed at a fixed potential. Chronoamperometry is a similar process of electrolysis conducted in a gas-tight two-compartment (anodic and cathodic) electrochemical H-cell with a Nafion-117 membrane as a proton exchanger or separator. Both compartments (anodic and cathodic) have the same electrolyte (0.5 M NaHCO_3_). Each compartment of the electrochemical H-cell had a 25 mL solution with approximately 20 mL headspace at a fixed potential. Before the electrolysis, the prototype H-type cell was flushed by bubbling N_2_ and CO_2_ for 30 min. During the electrolysis, the CO_2_ gas was continuously purged in a cathodic compartment with a constant flow rate, followed by the electrolysis time. The electrochemical setup is depicted in Fig. 2.

After completing the electrolysis, a small fraction of electrolyte was collected by a syringe and analyzed using GC-FID (6890 series GC system, Agilent Technologies Co.). The calculation of faradaic efficiency was figured out by performance (SI) for the electrochemical reduction of CO_2_ according to this equation.^47^i
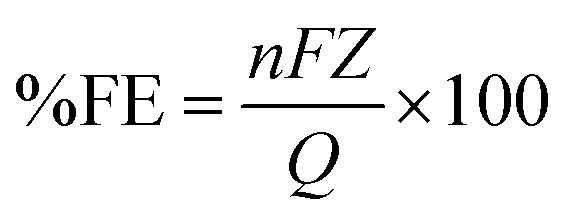
where, *n* = number of moles of the product. *Z* = numbers of electrons required to produce that given product. *F* = Faraday constant. *Q* = the total charge released.

## 3. Results and discussion

**Fig. 3:**
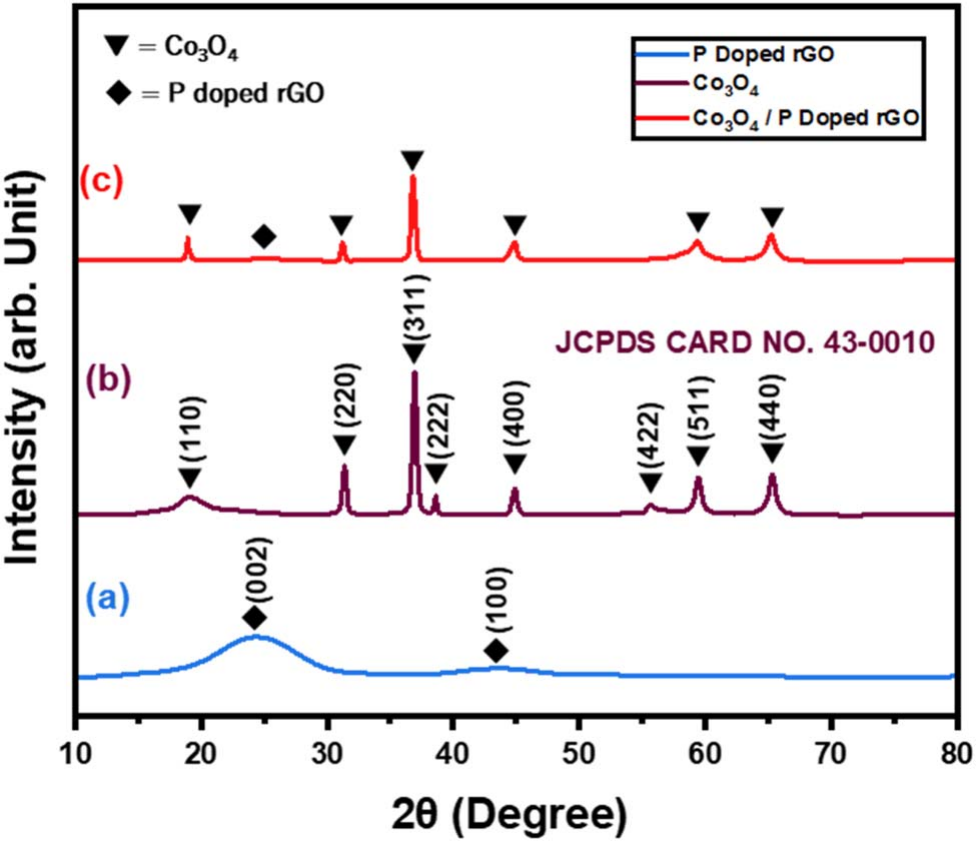
XRD of (a) P-doped rGO, (b) flower-like Co_3_O_4_, and (c) flower-like Co_3_O_4_/P-rGO.

Before evaluating the electrocatalytic activity for CO_2_ reduction, the structural, optical, and elemental properties of the synthesized catalysts were assessed using XRD, FESEM, EDX, Raman, and XPS. The crystallinity and purity of the synthesized materials were characterized by XRD analysis, as shown in Fig. 3.

Fig. 3(a) demonstrates that P-doped rGO exhibits a sharp diffraction peak at 2*θ* = 24.18° and 2*θ* = 43.5° corresponding to (002) and (100) planes, respectively. Ordinary GO displays a diffraction peak, 2*θ* = 10.6° for the (002) plane (Fig. S2).^48^ Upon chemical or thermal reduction, the oxygen-containing groups are removed, resulting in a broader peak at 2*θ* = 24.10° for rGO. However, the synthesized P-rGO material shows a diffraction peak at 2*θ* = 24.18°, indicating that the π-conjugated structure of graphene has been restored considerably by the production of rGO.^49^ Additionally, a weaker peak at 2*θ* = 43.5° corresponds to the (100) plane, attributed to the turbostratic band of disordered carbon materials.^50^

Fig. 3(b) presents peaks of 19.11°, 31.65°, 36.92°, 38.61°, 45.19°, 55.89°, 59.42°, and 65.3°, corresponding to the flower-like Co_3_O_4_ with crystalline planes (110), (220), (311), (222), (400), (422), (511), and (440), respectively. The XRD peaks for shape-controlled Co_3_O_4_ exhibit a strong correlation with the data from the JCPDS card no. 43-0010.^51^ This symbolizes that the pure shape controlled Co_3_O_4_ is formed without any impurities. The Fig. 3(c) describes the shape controlled Co_3_O_4_/P-rGO hybrid material consist the diffraction peak (2*θ*) of 19.11°, 31.65°, 36.92°, 45.19°, 59.42° and 65.3° represent the crystal plane (110), (220), (311), (400), (511) and (440) respective to shape controlled Co_3_O_4_ which was finely attributed with the JCPDS card no. 43-0010. Additionally, an extra diffraction peak was observed at 24.6° with the (002) plane, which was ascribed to disordered stacked graphitic sheets coordinated with the P-doped rGO XRD pattern that was earlier mentioned in Fig. 3(a).^52^ This indicates that all the diffraction peaks were assigned with the structural formation of flower-like Co_3_O_4_/P-rGO hybrid composite, similar to both JCPDS card no (43-0010) and card no (41-1487) for individual shape-controlled Co_3_O_4_ and P-rGO component.

The average crystalline size of synthesized P-rGO, Co_3_O_4_, Co_3_O_4_/P-rGO was calculated by the Debye Scherrer equation.^53^ Asii
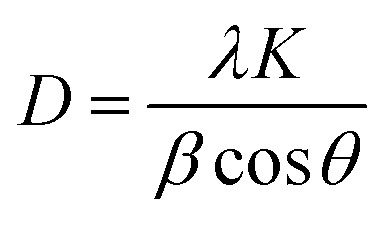
where, *K* = 0.9 is Scherrer's constant, *λ* is the wavelength of X-rays, *θ* is the Bragg diffraction angle *β* is the full width at half-maximum (FWHM) of the diffraction peak corresponding to the plane.

**Fig. 4:**
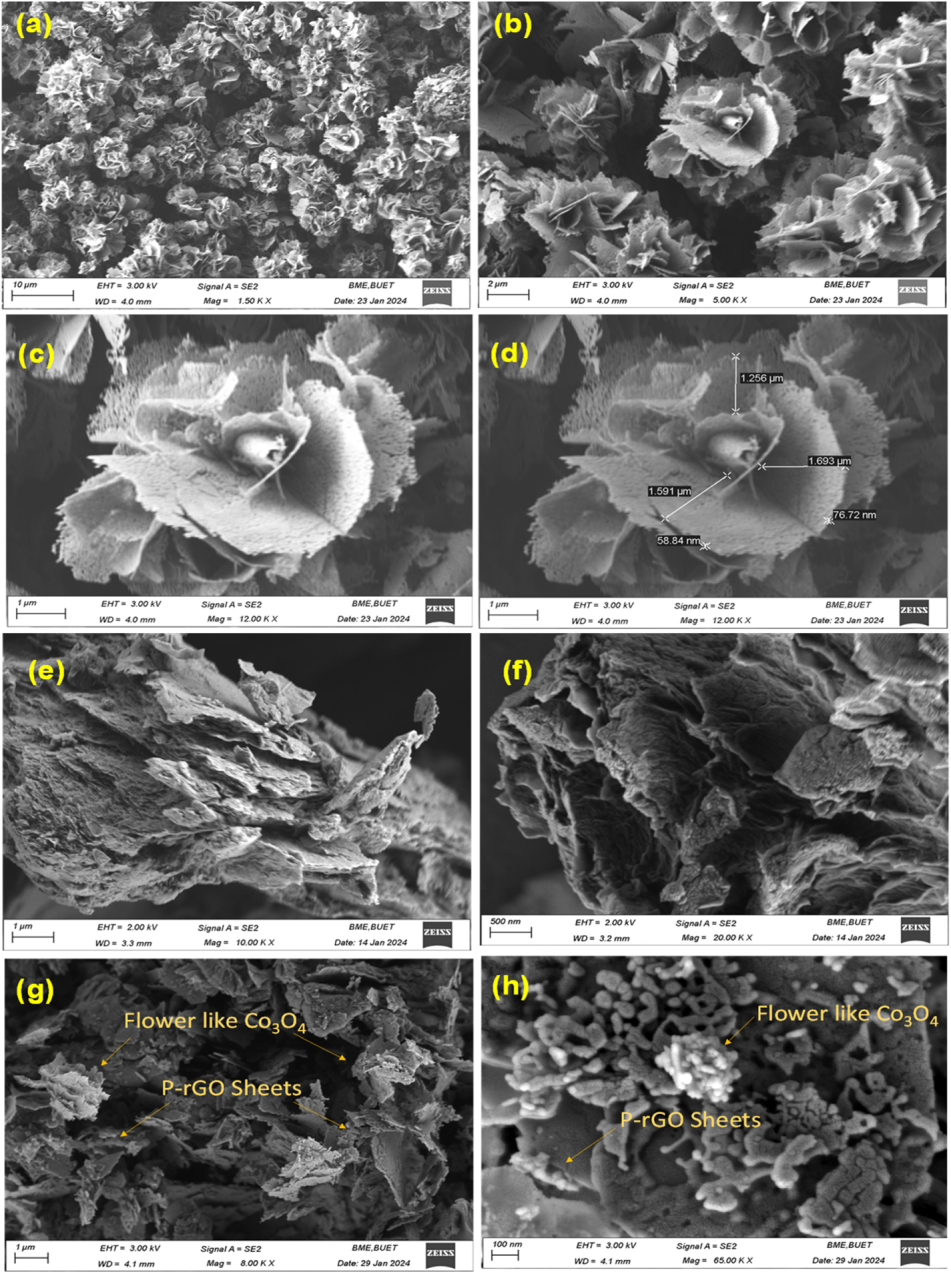
FESEM images of (a–d) flower-like Co_3_O_4_ (e and f) P-doped rGO (g and h) composite of Co_3_O_4_/P-rGO.

A distinct broadening of XRD peaks indicates that the material prepared is composed of a structure on the nanometer scale. The average crystalline size of GO, P-rGO, Co_3_O_4_, Co_3_O_4_/P-rGO is 4.54 nm (Fig. S2), 1.54 nm, 15.01 nm, and 14.79 nm, respectively. A slightly smaller crystallite size was observed for the hybrid Co_3_O_4_/P-rGO compared to pristine Co3O4, indicating the incorporation of P-rGO. Also, the interplanar spacing or *d*-spacing of synthesized materials is determined by Bragg's equation. The equation is,*nλ* = 2*d* sin *θ*iii
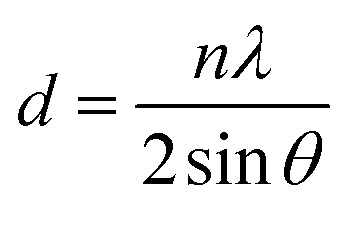
where, *n* is the order of the diffraction peak, *λ* is the wavelength of X-rays, *θ* is the Bragg diffraction in radians, and *d* is the interplanar distance or *d* spacing. The interlayer spacing of synthesized materials GO, P-rGO, Co_3_O_4,_ and Co_3_O_4_/P-rGO was found to be 0.61 nm, 0.28 nm, 0.26 nm, and 0.23 nm, respectively. This indicates a decrease in the shape controlled Co_3_O_4_/P-rGO hybrid material compared to pristine Co_3_O_4_ and P-rGO substrate. This is because the P-rGO aids in the segregation and dispersion of Co_3_O_4_ particles during the synthesis, facilitating the formation of the hybrid composite, as mentioned in the SEM images in Fig. 4(h).^54,55^

Surface morphology of the synthesized Co_3_O_4_, P-rGO, Co_3_O_4_/P-rGO materials was analyzed using FESEM, as shown in Fig. 4. Morphological attributes are influenced by crystallization, resulting in diverse structural shapes and sizes. Fig. 4(a–d) illustrates the shape-controlled Co_3_O_4,_ which exhibits a distinct flower-like microstructure. These structures were composed of relatively uniform, self-assembled sheets that form petal-like subunits. At higher magnification, the petal-like structure consists of thin micro-sheets, contributing to the flake-like texture of each petal. The average diameter of these flower-like Co_3_O_4_ microstructures is approximately 4 µm, with a size distribution ranging from 2 µm to 8 µm. The length of the petal-like sub-units, which extended into micro sheets, was exhibited on a micro scale. The average length of the petal-like subunits extending from the flower's center was 1.3 µm. As observed in Fig. 4(d), the petal-subunit length lies in the micrometer range, with the edges tapering to the nanometer scale. Fig. 4(e and f) depicts the morphology of P-doped rGO material, which exhibits crumpled, sheet-like structures resembling cabbage leaves. The wrinkled surface was attributed to the high-temperature hydrothermal treatment applied to the synthesized P-doped rGO from GO. The rough and multilayer texture confirms the successful incorporation of P into rGO structures. On the contrary, Fig. 4(g and h) represents the morphologies of flower-like Co_3_O_4_/P doped rGO hybrid material, where the flower-like Co_3_O_4_ is anchored on the layer-structured P-doped rGO sheets. Unlike the pristine Co_3_O_4_, the flower diameters and their petal subunits in the hybrid Co_3_O_4_/P-rGO composite are notably smaller, falling within the nanometer scale as shown in the higher magnification in Fig. 4(h). The size reduction is likely due to P atom doping, which modifies the charge density at the graphene surface, as confirmed by XRD analysis (Fig. S2).

Additionally, partially disrupted flower centers observed on the P-rGO sheets were likely due to the coordination of Co^3+^ ions with negatively charged phosphorous-containing functional groups on the rGO sheets. During the ultrasonication process, Co^2+^ ions were oxidized to Co^3+^ by oxygen-containing groups, leading to the crystallization of Co_3_O_4_ particles that subsequently nucleated and anchored onto the P-rGO sheets.^56^ While this mechanism aligns with the observed particle size and distribution, it should be considered a speculative interpretation rather than a confirmed mechanism, given the known role of defect-rich, heteroatom-doped carbon materials in providing nucleation sites for metal species.

**Fig. 5:**
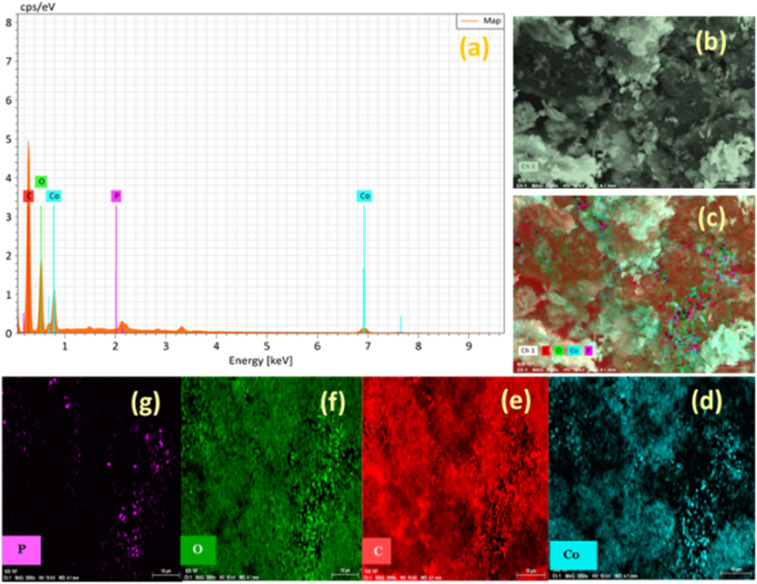
EDX analysis of Co_3_O4/P-rGO; (a) elemental spectrum (b and c) EDX mapping of selected area of identifying Co, P, C, O elements; (d) Co elements (e) C elements (f) O element (g) P element.

The EDX analysis of Co_3_O_4_, P-doped rGO, and Co_3_O_4_/P-rGO materials was presented in Fig. 5. The EDX spectra provide qualitative and quantitative information about the chemical composition of a material.^56^ The Co_3_O_4_ sample showed Co and O atom percentages of 26.12% and 73.88%, respectively. EDX mapping confirmed the exclusive presence of Co and O, with no detectable impurities (Fig. S3). The P-rGO material contained C(81.05%), O (17.70%), and P(1.25%) in P-rGO, respectively (Fig. S4), confirming successful doping of P onto the rGO matrix. Although EDX does not reveal the information of chemical bonding, it gives the elemental composition of materials in mass and atom percentage.^57^ EDX spectra of the Co_3_O_4_/P-rGO hybrid showed the presence of Co, C, O, and P with atom percentages of 11.03%, 48.52%, 39.31% and 1.01%, respectively. Elemental mapping confirmed the spatial distribution of these elements, where Co derived from the Co_3_O_4_ anchored on the surface of the P-rGO, C originated from the rGO, O from both the rGO and the Co_3_O_4_ materials, and P from the doped rGO, confirming the material's high purity and successful synthesis.

**Fig. 6:**
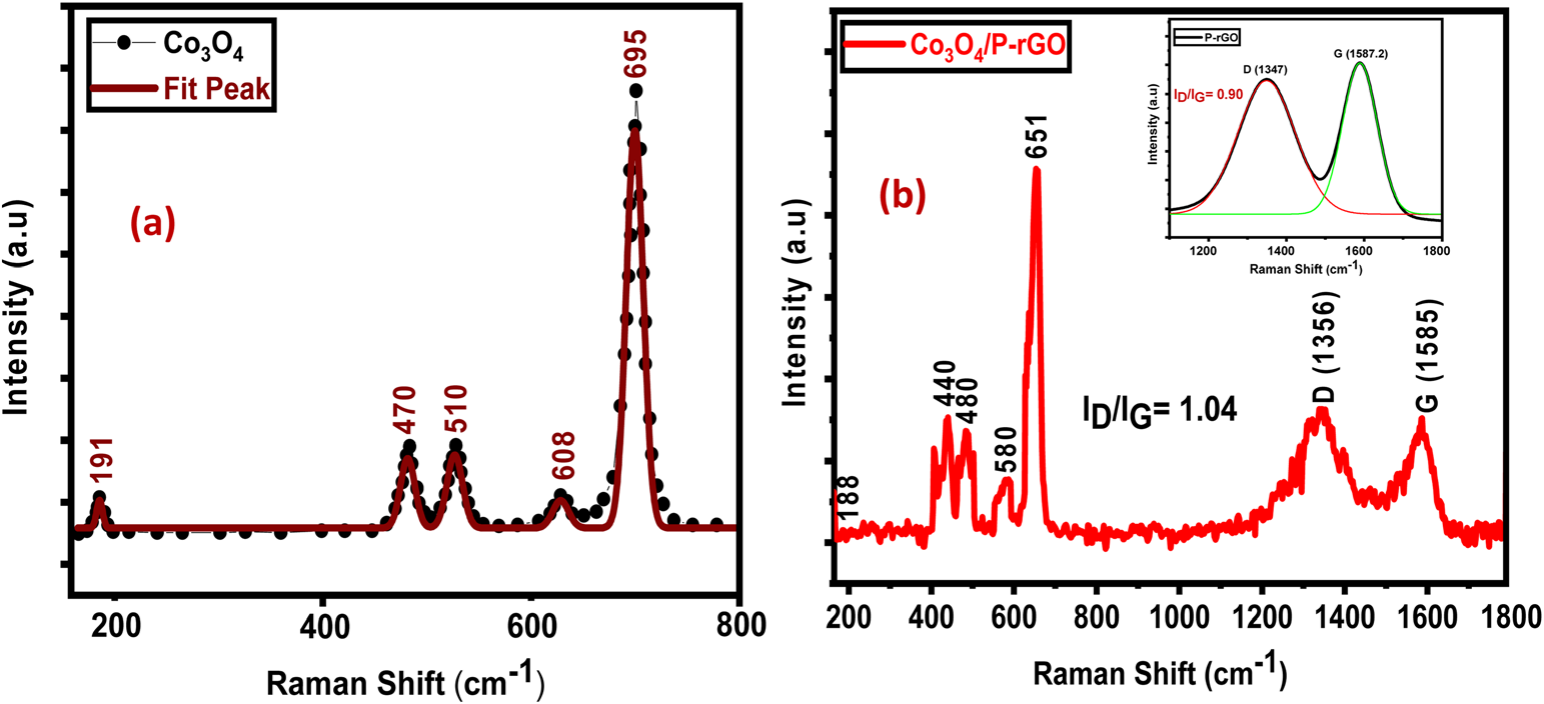
Raman spectra of (a) flower-like Co_3_O_4_ (b) flower-like Co_3_O_4_/P-rGO.

The Raman spectra of Co_3_O_4_ and the Co_3_O_4_/P-rGO composite are shown in the Fig. 6(a) and (b), respectively, while the spectra of GO, rGO, and P-doped rGO are provided in Fig. S5. Five characteristic vibrational modes were observed in Fig. 6(a) for pristine Co_3_O_4_ at approximately 191, 470, 510, 608 and 695 cm^−1^, which are in good agreement with reported Raman-active modes of spinel Co_3_O_4_. The intense peak at 695 cm^−1^ corresponds to the A_1_g mode, associated with the symmetric stretching of the oxygen atom in CoO_6_ octahedra, confirming the formation of a well-crystallized spinel structure. The Raman spectra of GO, rGO, and P-rGO display the characteristic D (1350) and G (1580) bands. The calculated *I*_D_/*I*_G_ ratios for GO, rGO, and P-rGO are 1.37, 1.27, and 0.90, respectively (Fig. S5). The progressive decrease in *I*_D_/*I*_G_ ratio from GO to rGO indicates a partial restoration of the sp^2^ carbon network and a reduction in structural defects during the reduction process. A further decrease for P-rGO indicates the phosphorous doping promotes structural reorganization resulting in an increase in the average size of sp^2^ domains and a more continuous conjugated carbon framework.

For Co_3_O_4_/P-rGO, the coexistence of Co_3_O_4_ vibrational modes and the D and G bands confirms the successful formation of the hybrid structure, as shown in Fig. 6(b). The *I*_D_/*I*_G_ ratio of the hybrid Co_3_O_4_/P-rGO is 1.04, which lies between those of Co_3_O_4_ and P-rGO. This enhancement in defects can be attributed to strong interfacial interactions between Co_3_O_4_ and the P-rGO, which induce structural distortion and generate additional defect sites. Additionally, a slight red shift in the D and G bands is observed after composite formation, indicating electronic interaction between Co_3_O_4_ and the P-rGO support. This shift can be attributed to charge transfer effects and lattice strain induced by the anchoring of Co_3_O_4_ onto the P-rGO surface.

**Fig. 7:**
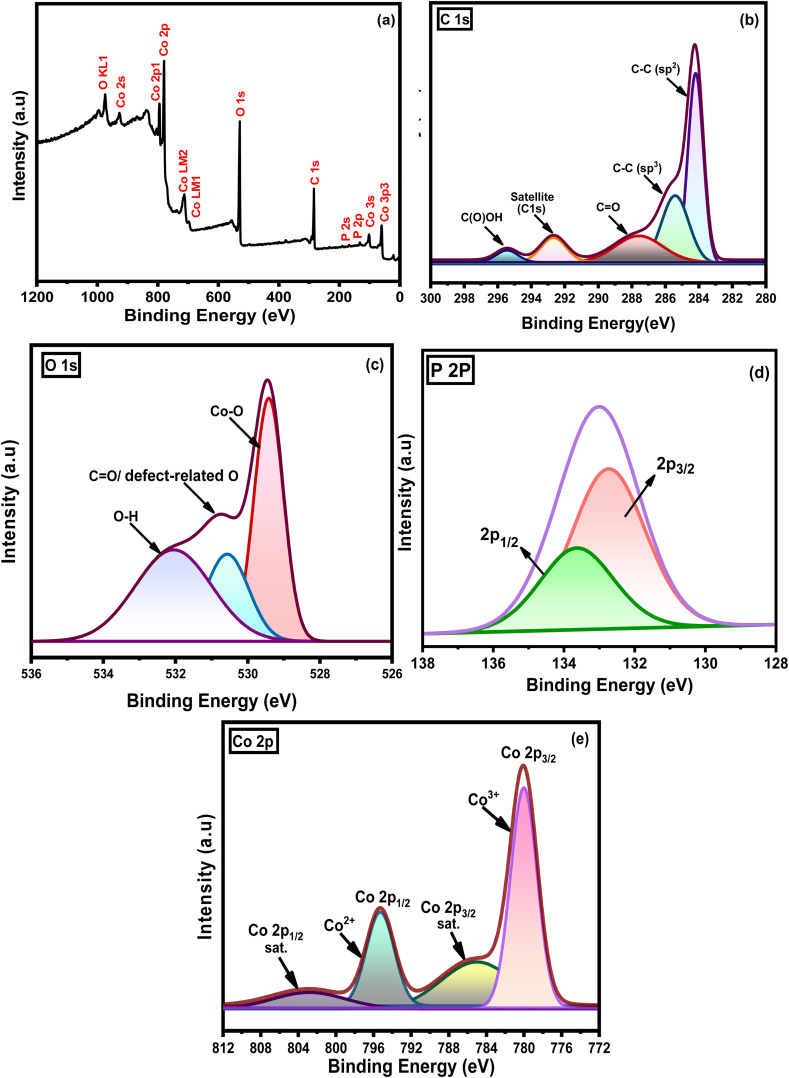
XPS images of (a) survey spectra of Co_3_O_4_/P-rGO; (b–e) survey scan of (b) C 1s, (c) O 1s, (d) P 2p, and (e) Co 2p.

X-ray photoelectron spectroscopic (XPS) analysis was performed to determine the surface elemental composition, electronic structure, and deconvoluted spectra of Co_3_O_4_/P-rGO hybrid material. Fig. 7(a) demonstrates the survey spectrum of the prepared Co_3_O_4_/P-rGO hybrid material consisting of C, O, P, and Co elements. The rGO sheets were accumulated in a synthesized Co_3_O_4_/P-rGO hybrid material, which was also confirmed by the survey spectrum in Fig. 7(a) and the asymmetric deconvolution of the carbon C(1s) spectra of Fig. 7(b). The C(1s) spectra exhibit five distinct peaks of deconvoluted positioned at 284, 285, 288, 292 and 295 eV which are attributed to sp^2^ hybridized C–C in aromatic rings, epoxy/ether group (C–O), sp^3^ hybridized C–C/C–H bonds and a shake-up satellite peak (π → π*) as a characteristic of aromatic C structures and carboxylic group (O–C=O), respectively.^58,59^ These spectral features indicate a shift in the binding energies of oxygenated functional groups, along with the successful incorporation of P dopant in the prepared hybrid material. The observed shift of these oxygenated groups creates a strong interaction to anchor the shape-controlled Co_3_O_4_ with the functional groups of P-rGO material.^60^ Furthermore, the deconvoluted peak of oxygen O(1s) from Fig. 7(c) yields three peaks centered at 529, 530, and 532 eV corresponding to the Co–O bond in the metal oxide lattice, oxygen-deficient sites (vacancies) or C=O, and chemisorbed oxygen anions or oxygen related to OH ions, respectively.^61^ The binding energies of O(1s) confirmed that the defect sites are formed in the synthesized flower-like Co_3_O_4_/P-rGO hybrid material. The high‑resolution P 2p XPS spectrum exhibits two characteristic peaks at approximately 132 and 133 eV, corresponding to P 2p_3/2_ and P 2p_1/2_, respectively. These peaks are attributed to phosphorus atoms in the phosphate group, as shown in Fig. 7(d).^62,63^ The XPS spectrum in Fig. 7(e) reveals that the two main peaks positioned at 780, and 795 eV respectively consist of CO_2p_3/2__^3+^ and CO_2p_1/2__^2+^ oxidation state with two satellite peaks located at 785 and 803 eV.^64^ The chemical state is also determined by measuring the difference between two spin separation energies between CO_2p_3/2__^3+^ and CO_2p_1/2__^2+^. Deconvolution of these peaks indicates contributions from both Co^3+^ and Co^2+^ species, consistent with the coexistence of mixed valence states in the Co_3_O_4_/P-rGO composite. For instance, the spin–spin splitting between CO_2p_3/2__^3+^ and CO_2p_1/2__^2+^ is 15 eV signifies low spin Co^3+^ species, are present in composite phase.^65^ Additionally, the two corresponding satellite peaks positioned at 803 and 785 eV for Co 2p_1/2_ and Co 2p_3/2_, correspond to the Co^2+^ species. That means, a portion may be attributed to reducing Co^3+^ to Co^2+^ and creating oxygen vacancies.

**Fig. 8:**
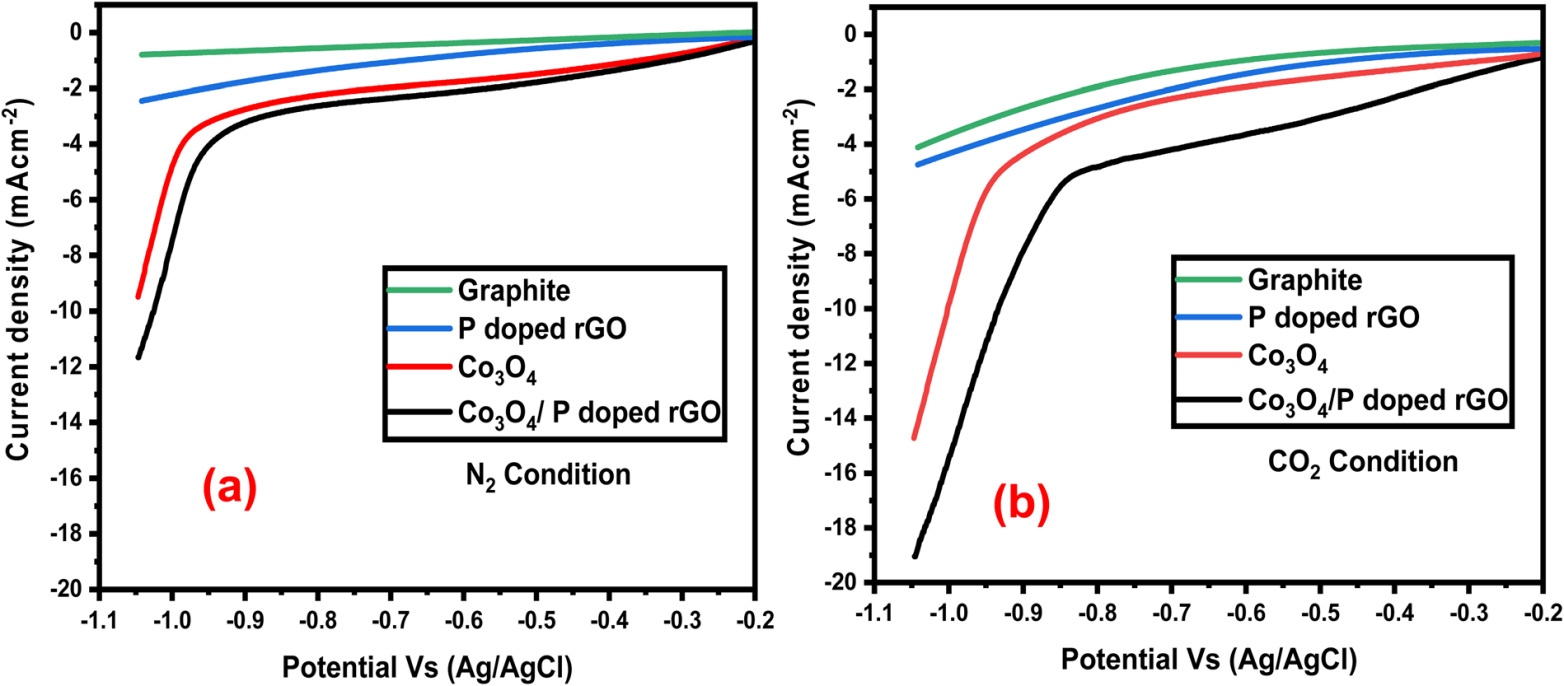
LSV of prepared catalyst (a) N_2_ condition, (b) CO_2_ condition, in 0.5 M NaHCO_3_ aqueous solution at 30 mV s^−1^ scan rates.

Linear sweep voltammetry (LSV) was conducted to evaluate the electrocatalytic reduction of CO_2_ on the prepared cathode electrodes. LSV was carried out in both N_2_ and CO_2_ saturated 0.5 M NaHCO_3_ aqueous solution as an electrolyte at a scan rate of 30 mV s^−1^ in a typical three electrode system, where Ag/AgCl (3 M) was used as a RE, platinum wire as a CE, and the prepared catalyst was used as the modified WE. The pH was maintained in N_2_, and CO_2_-saturated 0.5 M NaHCO_3_ aqueous solutions were 8.5 and 7.5, respectively. Each system was cycled 15 times over a potential range of −0.2 to −1.05 V at a scan rate of 30 mV s^−1^ to stabilize the electrode response. Fig. 8(a) displays relatively low reduction currents under N_2_, while Fig. 8(b) highlights a substantial increase under CO_2_, confirming the catalyst's activity in CO_2_ reduction. The bare graphite electrode showed a negligible current in N_2_, but a significant increase in current was observed from −0.6 V in the presence of CO_2_. By modifying the graphite electrode using P-rGO, flower-like Co_3_O_4_ and flower-like Co_3_O_4_/P-rGO catalyst, the cathodic current densities under N_2_ were −1.04, −2.42, −9.58 and −11.70 mA cm^−2^, respectively, due to proton/water reduction. In contrast, under CO_2_-saturated conditions, the current was increased with the P-rGO, Co_3_O_4,_ and Co_3_O_4_/P-rGO catalyst by modifying the graphite electrode. From Fig. 8(b), the decreasing current in CO_2_ conditions was found to be −3.82, −5.75, −14.77, and −19.15 mA cm^−2^, respectively, for graphite and graphite modified with P-rGO, Co_3_O_4_, and Co_3_O_4_/P-rGO cathode electrodes. Fig. 8(a) and (b) shows that both the set data of N_2_ and CO_2_ conditions with a single graphite electrode and the modified electrode with P-rGO, Co_3_O_4_, and Co_3_O_4_/P-rGO catalyst had a drastic change in reduced current. These observations indicate that the presence of CO_2_ significantly enhances the reduced current, with the current densities increasing by factors of approximately 2, 3, 5, and 8 for P-rGO, Co_3_O_4_, and Co_3_O_4_/P-rGO catalysts, respectively, compared to N_2_ conditions. Thus, the enhancement of reductive current indicates that the catalytic activity of the modified electrode using P-rGO, Co_3_O_4_, and Co_3_O_4_/P-rGO catalysts can reduce CO_2_. The LSV data clearly demonstrate the superior electrocatalytic performance of the synthesized flower-like Co_3_O_4_/P-rGO composite, outperforming its individual entities, indicating that a synergistic effect between the two materials is effective for the electrocatalytic reduction of CO_2_.

**Fig. 9:**
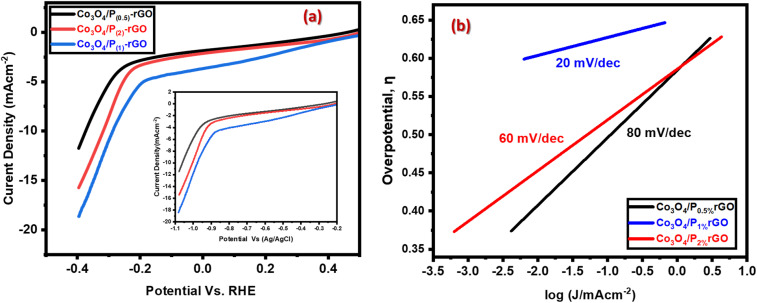
Comparison of the CO_2_RR performance (a) LSV analysis (b) Tafel plots for different amounts of dopant composition to hybrid materials.

The catalyst utilizes a metal–organic framework-based WE, featuring flower-like Co_3_O_4_ as the metal framework and rGO as the organic framework. Following the phosphorus doping into rGO substrates and binding with Co_3_O_4_, the catalytic performance of the resulting Co_3_O_4_/P-rGO hybrid structure has greatly improved by varying the amount of P that has been doped. LSV was used to investigate the catalytic performance of Co_3_O_4_/P-rGO hybrids with varying P doping ratios. The P doping levels in the heterostructures were precisely adjusted to 0.5%, 1%, and 2%. Fig. 9(a) and (b) show that an increase in the P doping ratio led to a higher reduction current, with increments of −11.52, −18.51, and −15.36 mA cm^−2^, and Tafel slopes of 80, 60, and 20 mV dec^−1^, respectively. Catalytic performance was evaluated by comparing the potentials at which specific current densities were achieved according to the RHE, as shown in Fig. 9(a). The kinetic parameters of CO_2_ reduction in different electrodes were calculated according to the given equations,^66^*η* = *a* + *b* log *i*where, *η* is the overpotential (volts), *i* is the current density (mA cm^−2^), *a* is the Tafel constant (often related to the exchange current density) and *b* is the Tafel slope (mV per decade).

In Fig. 9(b), the catalyst was prepared using a metal–organic framework-based working electrode, featuring flower-like Co_3_O_4_ as the metal framework and rGO as the organic framework. Phosphorus doping into the rGO substrate and its interaction with Co_3_O_4_ significantly enhanced the catalytic performance of the resulting Co_3_O_4_/P-rGO hybrid. The P doping levels were precisely controlled at 0.5%, 1%, and 2%. The catalytic performance of the Co_3_O_4_/P-rGO hybrids was investigated using LSV, as shown in Fig. 9(a). Increasing the P content from 0.5% to 1% increased the reduction current from −11.52 to −18.51 mA cm^−2^, while further increasing P to 2% led to a slight decrease to −15.36 mA cm^−2^.

Tafel plots in Fig. 9(b) were used to evaluate the reaction kinetics. The Tafel slope decreased sharply from 80 mV dec^−1^ at 0.5% P to 20 mV dec^−1^ at 1% P, indicating accelerated electron transfer and enhanced catalytic kinetics. However, at 2% P, the Tafel slope increased again to 60 mV dec^−1^, likely due to overdoping that caused partial active-site blockage or structural distortion. A smaller Tafel slope corresponds to faster kinetics, enabling the catalyst to achieve higher current densities under moderate applied potentials. The Tafel slope provides a reliable measure of kinetic efficiency without the need for explicit overpotential calculations.^66,67^ Based on these analyses, the Co_3_O_4_/P_(1%)_-rGO hybrid exhibited the lowest Tafel slope and best overall CO_2_ reduction performance, and was therefore selected for further characterization (XRD, FESEM, XPS, and EDX) and electroreduction studies. The Tafel slope shows a significant dependence on the P doping level. Increasing the P content from 0.5% to 1% results in a sharp decrease in the Tafel slope from 80 mV dec^−1^ to 20 mV dec^−1^, indicating enhanced catalytic kinetics. However, a further increase to 2% lead to an increase in Tafel slope again to 60 mV dec^−1^. A smaller Tafel slope signifies accelerated electron transfer and faster catalytic kinetics, allowing the catalyst to reach high current densities more readily under moderate applied potentials.^67^ These findings demonstrate that a 1 wt% P doping yields the lowest absolute Tafel slopes, which is the smallest (20 mV dec^−1^), offering the best CO_2_ reduction performance and excellent stability. On the other hand, the performance drop at 2% P doping may be due to overdosing, leading to active-site blockage or structural distortion. Based on this optimization, the hybrid flower-like Co_3_O_4_/P_(1%)_-rGO structure was selected for further material characterization (XRD, FESEM, XPS, and EDX) and CO_2_ electroreduction applications.

**Fig. 10:**
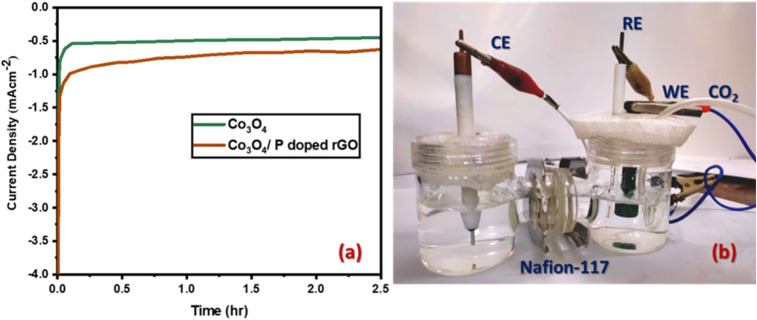
Chronoamperometry response for CO_2_ electroreduction for (a) prepared Co_3_O_4_ (green line) and Co_3_O_4_/P-rGO (brown line) catalyst at −0.62 V in H-type prototype; (b) setup for chronoamperometry process.

Since both water and CO_2_ can be reduced under such conditions, LSV alone is insufficient to assess electrocatalytic activity for CO_2_ reduction.^68^ Therefore, potentiostatic electrolysis or chronoamperometry was performed in a CO_2_-saturated 0.5 M NaHCO_3_ aqueous solution (pH value 7.5) at a constant of −0.62 V in an H-type electrochemical cell for 2.5 hours. The resulting liquid-phase products were analyzed using gas chromatography-mass spectrometer (GC-MS, Clarus 680). The selection of the applied potential for CO_2_ conversion was guided by Tafel plot analysis, which identified an apparent equilibrium potential and the kinetically favorable region for CO_2_ reduction.^69^ CO_2_ was continuously purged at a flow rate of 10 mL min^−1^ in the cathodic chamber, with a regulator-maintained pressure of 362 psi (0.06 bar) from the gas cylinder, which was separated from the anodic chamber by a Nafion-117 membrane, as shown in Fig. 10(b). A constant voltage (−0.62 V) was applied for different modified electrodes. The chronoamperometry was taken in an H-type electrochemical cell using Ag/AgCl as RE. From LSV data, a notable increase in current density was observed beginning at −0.6 voltage (*vs.* Ag/AgCl) in LSV data for the flower-like Co_3_O_4_ electrode. Furthermore, after analyzing the Tafel plot (Fig. S7) for CO_2_ reduction, the −0.6 V selected for product selectivity is adjusted to −0.62 to ensure an adequate driving force for CO_2_ reduction while remaining within the kinetically favorable region.

Potentiostatic measurements were conducted on Co_3_O_4_ and Co_3_O_4_/P-rGO electrode, prepared by modifying a graphite substrate. A stable current density was exhibited, approximately −0.5 mA cm^−2^, while the flower-like Co_3_O_4_/P-rGO hybrid material maintained a consistent current density of −0.8 mA cm^−2^ at the same potential. The total charge released, or the number of electrons introduced during this chronoamperometric process, which is essential for estimating the faradaic efficiency, was determined by integrating the current–time (*I*/*t*) curve over the course of the experiment.^70^ The charges released for the Co_3_O_4_ and Co_3_O_4_/P-rGO modified electrode for a 2.5-hour electrolysis time were found to be 1.85C and 3.65C, respectively. Comparing the current density and the amount of charge released for both the Co_3_O_4_ and Co_3_O_4_/P-rGO electrodes, at a fixed potential, both the cathodic current density and the released charge amount increase. This indicates that the synthesized Co_3_O_4_ and Co_3_O_4_/P-rGO modified electrodes exhibit effective cathodic behavior. The synthesized Co_3_O_4_ and Co_3_O_4_/P-rGO materials worked as a cathode and showed very stable operation for the electrocatalytic reduction of CO_2_ under a constant applied potential.

**Fig. 11:**
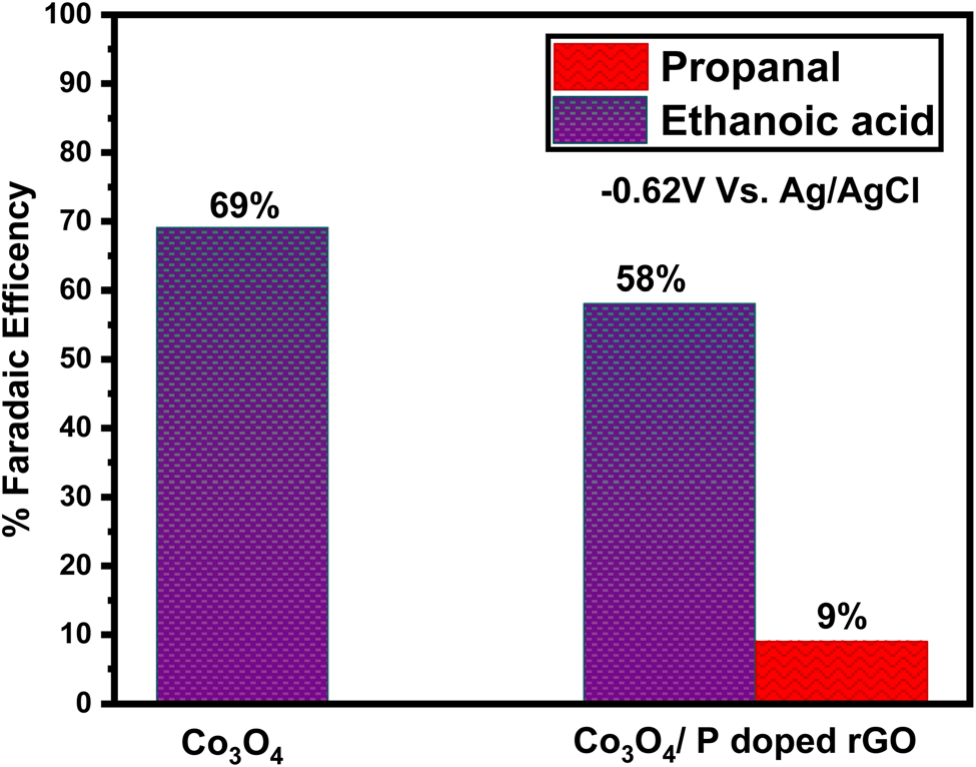
Study of the faradaic efficiency of produced product *versus* electrolysis voltage as prepared catalyst Co_3_O_4_ and Co_3_O_4_/P-rGO in 0.5 M NaHCO_3_ aqueous solution.

After completing the electrolysis, the resultant liquid products were analyzed using a Clarus 680 gas chromatography-mass spectrometer (GC-MS). The faradaic efficiency of the produced product was determined using a modified graphite electrode with Co_3_O_4_ and Co_3_O_4_/P-rGO catalysts under a constant applied voltage of −0.62 V, as illustrated in Fig. 11. The current density from the chronoamperometric (Fig. 10) measurements reflects sustained product formation over a long period of electrolysis. Although hydrogen evolution is recognized as a competing reaction in CO_2_ reduction systems, accurate quantification of H_2_ was not feasible because of limitations in gas collection and trapping during electrochemical measurements. The Co_3_O_4_ catalyst achieved a faradaic efficiency of 69% for ethanoic acid (SI), which was confirmed by GC-MS. In contrast, the hybrid Co_3_O_4_/P-rGO yielded a product mixture of ethanoic acid and propanal, with faradaic efficiency of 58% and 9%, respectively (SI). As the electroreduction of CO_2_ is pH sensitive, for the detection of electrolyte from GC-MS, the pH was found 7.1 ± 0.2.

As shown in Fig. 11, the use of Co_3_O_4_ alone predominantly yields a single liquid derivative, ethanoic acid, at 69% faradaic efficiency. However, when the Co_3_O_4_ and P-rGO were hybridized, the product selectivity changed. For the composition of flower-like Co_3_O_4_/P-rGO, the %FE_CH_3_COOH_ is decreased to 58% from 69% of the individual flower-like Co_3_O_4_ catalyst. Also, propionaldehyde or propanal is observed, with a faradaic efficiency of 9%. These findings indicate that the P-rGO materials give an effective electron transport capacity and facilitate the proton-coupled with the multielectron transfer of CO_2_ electroreduction reactions for the hybrid flower-like Co_3_O_4_/P-rGO catalyst. Ethanoic acid is the primary product and dominant for the catalyst of both Co_3_O_4_ and Co_3_O_4_/P-rGO materials at the same voltage. Compared to pristine Co_3_O_4_ catalyst, the Co_3_O_4_/P-rGO hybrid exhibited a decreased faradaic efficiency (%FE_CH_3_COOH_), while the formation of propanal was observed with a notable faradaic efficiency (%FE_C_2_H_5_CHO_) due to the synergistic effect between Co_3_O_4_ on P-rGO materials.

**Fig. 12:**
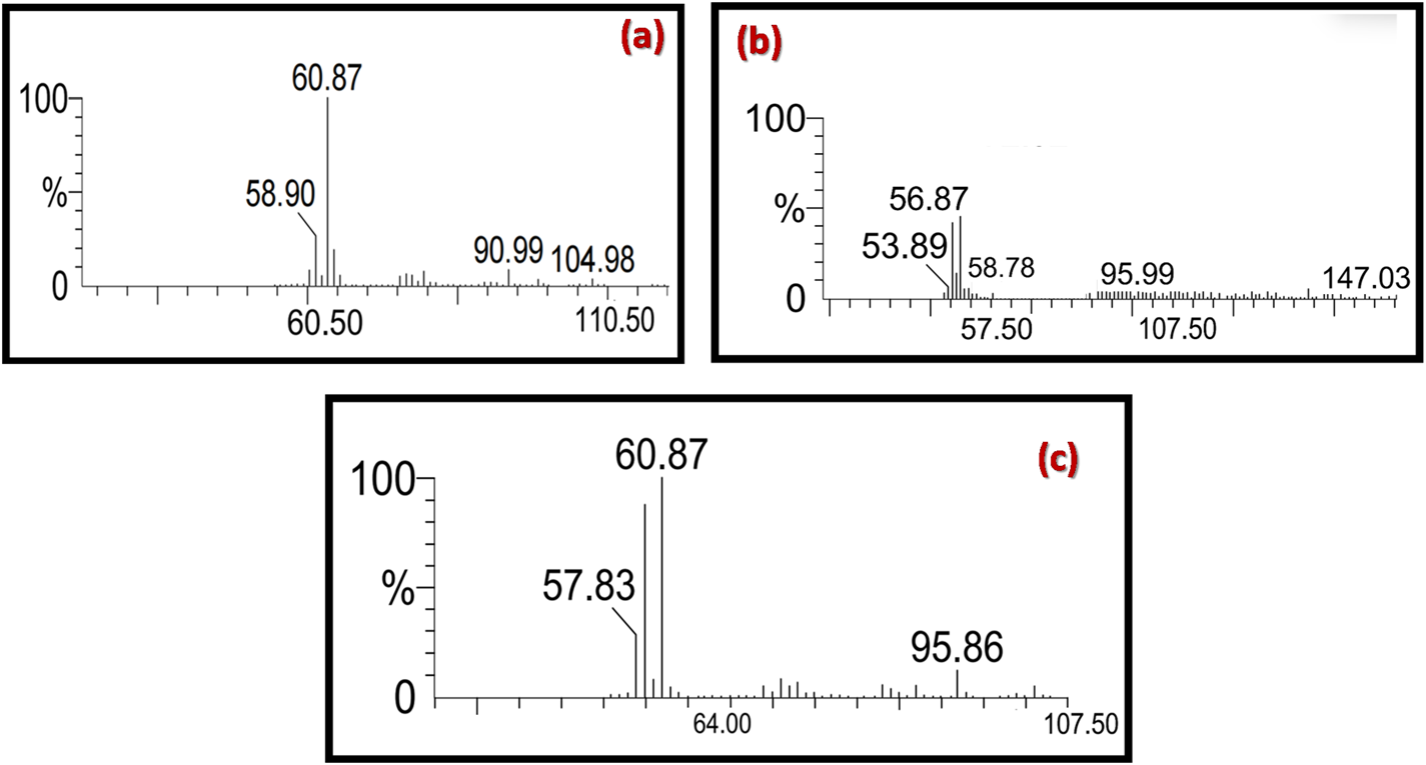
GC-MS spectra of electrolyte solution after 2.5 h chronoamperometry *vs.* Ag/AgCl, pH 7.5 (a and b) ethanoic acid (c) propanal.

To verify the liquid-phase product formed by the electrocatalytic reduction of CO_2_, GC-MS analysis was performed of the liquid electrolyte after electrolysis. Accurate product identification is essential for determining the effectiveness of the electrochemical method, including the catalyst, electrolyte, or overall reaction conditions. The mass spectra of the detected analytes were matched with the NIST spectral library (Table S2), and the quantification of liquid product was then performed by correlating the peak area with the calibration curve of the corresponding standard compounds (Fig. S8). As previously mentioned, ethanoic acid was detected as a major product when using a flower-like Co_3_O_4_ electrocatalyst. This was confirmed by the GC-MS spectrum shown in Fig. 12(a). The resulting GC-MS spectra in Fig. 12(a) consist of a base peak, which is the most abundant peak, and the characteristic fragments at *m*/*z* = 60.87 correspond to the acetic acid (CH_3_COOH^+^).^71^ Additionally, it has a molecular ion peak and characteristic fragments at *m*/*z* = 58.90 for the CH_3_COO^−^ fragment, along with an isotopic peak at *m*/*z* = 61, corresponding to ^13^CH_3_COOH^+^ or CH_3_^13^COOH^+^.^72^ In contrast, both ethanoic acid and propanal were also identified for the catalytic reduction of CO_2_ using the flower-like Co_3_O_4_/P-rGO hybrid materials as shown in Fig. 12(b and c). The GC-MS spectrum in Fig. 12(b) attributed a molecular ion peak at *m*/*z* = 60.87, consistent with acetic acid (CH_3_COOH^+^), further validating its presence in the electrolyte. Meanwhile, the spectrum in Fig. 12(b) confirms the formation of propanal, with a base ion peak appearing *m*/*z* = 56.87, which contributes to the mass fragments of C_3_H_5_O^+^ and a molecular ion peak at *m*/*z* = 58 for C_3_H_6_O^+^.^73^ The reduced intensity of this molecular ion peak in Fig. 12(b) indicates the coexistence of propanal in the electrolyte mixture.

**Fig. 13:**
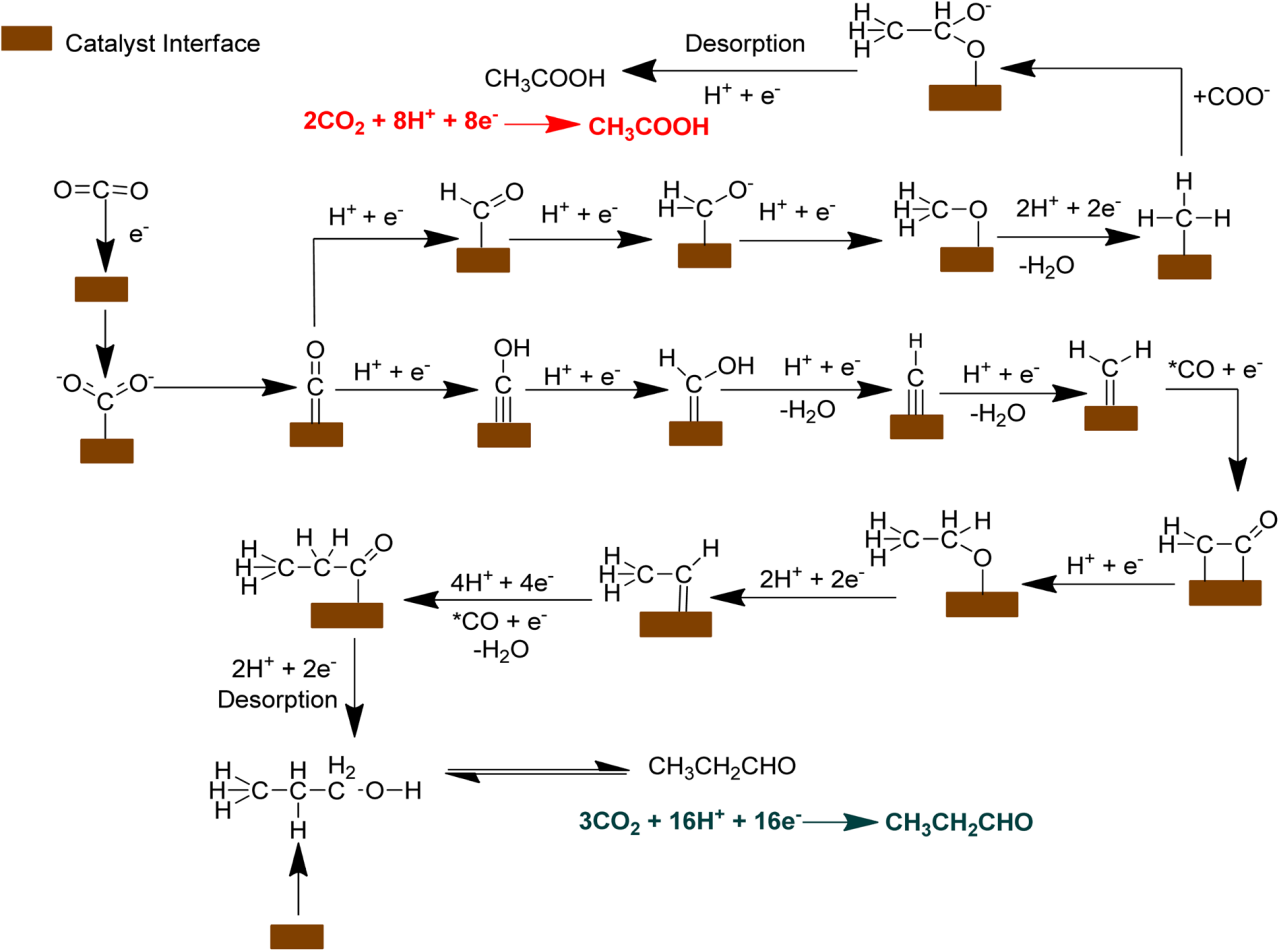
Possible reaction pathways for the electrocatalytic CO_2_ reduction to ethanoic acid (CH_3_COOH) and propanal (CH_3_CH_2_CHO).

Fig. 13 illustrates the electrocatalytic pathway for producing ethanoic acid and propanal from CO_2_ reduction. CO_2_ is reduced *via* proton-coupled electron transfer (PCET) steps. It undergoes reduction to form higher carbon selective C_2_ and C_3_ products. Acetic acid originates through the interaction between the CO_2_˙^−^ radical anion and the reduced (–CH_3_) species adsorbed on the catalyst's surface.^74^ The CO_2_˙^−^ radical anion first forms formaldehyde (HCHO), which can occour with or without a catalyst. The reaction proceeds with the reduction of CO_2_˙^−^, which either weakly adsorbed on the electrode surface or undergoes proton attack.^75^ After adsorption, the C–O bond breaks, releasing a water molecule and forming an –CH_2_O intermediate. Further reduction produces the –CH_3_ species, which serves as a precursor for acetic acid, occurring in a highly reducing environment, where electrons are supplied by the anode through an external circuit and the protons arrive at the cathode *via* the Nafion-117 membrane, which is in direct contact with the electrocatalyst. In the final stage, the C–O bond breaks in two stages, releasing water and forming ethanoic acid. This happens either *via* nucleophilic attack by non-adsorbed CO_2_˙^−^ or through the reaction between the adsorbed –CH_3_ species and CO_2_˙^−^ at a nearby catalytic site.^76^ Conversely, in the formation of propanal, the C–C–C bond formation is the key step for C_3_ product. It would occur in two stages: C–C bond formation and post C–C coupling steps.^77^Fig. 13 demonstrates the potential pathways either the direct formation of the CO dimer, or CO reacting simultaneously with H^+^ and e^−^ to generate the HCOH intermediate. While another pathway involves the protonation of CO to form –CHO, which is selective for methane and glyoxal products. However, at a very negative overpotential, CO dimerization is reportedly difficult.^78^ As a result, CO is more likely to reduce to –COH, which then forms the HCOH intermediate. Subsequently, through proton–electron-coupled transfer and water elimination, the HCOH intermediate is transformed into –CH_2_. The proton-coupled electron transfer serves as the rate-determining step in this pathway which implies that the process is influenced by pH. For C–C bonding formation, CO is inserted into –CH_2_. It is found that the charged water layer could stabilize the CO dimer, and without charged water layer, the CO dimer formation is energetically prohibited. Due to CO insertion, the –CH_2_ is then shifted to H_2_CCO^−^.^79^ After the post coupling step of C–C, it is shown that protonation of the H_2_CCO^−^ intermediate results in CH_3_CH_2_O^−^, which are then further reduced to CH_3_CH. Then CH_3_CH group is subsequently reduced to form CH_3_CO by the addition of hydrogen and electron transfer, breaking molecular bond, leading to the formation of the acetyl group. The reduction process may involve the conversion of CH_3_CH which is further reduced to CH_3_CO, acting as a precursor for C_3_ products. The precursor CH_3_CO is further converted to CH_3_CHCHO^−^ due to the CO insertion, proton–electron-coupled transfer and water elimination.^80^ Protonation into CH_3_CHCHO^−^ intermediate converted CH_3_CH_2_CH_2_OH and desorb from the catalyst. As previously mentioned, the electroreduction of CO_2_ is pH responsive, under specific conditions-including voltage, anodic materials, pH (7.1 ± 0.2) and the concentration of CO_2,_ the CH_3_CH_2_CH_2_OH is oxidized to CH_3_CH_2_CHO.

Materials like platinum, gold, or carbon-based electrodes are commonly used for alcohol oxidation.^81^ In this case, the platinum wire is used as an anode, which is highly effective for oxidizing alcohols to aldehydes, with high activity and stability. It is reasonable to predict that propanol is oxidized to propanal, releasing protons and electrons. This transformation occurs as the catalyst selectively promotes the dehydrogenation of the alcohol group to form the aldehyde.

Table 1 summarizes the performance of numerous Co_3_O_4_-based catalysts in the electrochemical reduction of carbon dioxide, highlighting the influence of catalyst morphology, composition, electrolyte, applied potential, and their impact on product selectivity, faradaic efficiency, and operational stability. The CO_2_ electroreduction performance of various Co_3_O_4_-based catalysts was compared (Table 1). Co_3_O_4_/N-GO nano-cubes and atomic Co layers showed the highest formate (HCOOH) faradaic efficiencies (83% and 90%), while ultrathin Co_3_O_4_ had moderate formate selectivity (60%) with longer stability (20 h). Flower-like Co_3_O_4_ and Co_3_O_4_/P-rGO favored acetate (CH_3_COOH) formation (69% and 58%), but with shorter stability (∼2.5 h). Other morphologies promoted CO, ethanol, or ethylene production. This shows that morphology and support strongly affect selectivity, and nano-engineering strategies like rGO supports can enhance both product formation and stability. It can be observed that flower-like Co_3_O_4_/P-rGO achieves notable acetate production through synergistic effects of the support, despite moderate stability.

**Table 1:** CO_2_ electroreduction performance of various Co_3_O_4_-based catalysts

Catalyst	Electrolyte	Shape	Potential	Product (%FE)	Stability	Ref.
Co_3_O_4_	0.1 M TBAPF_6_ in ACN + 1% vol H_2_O	Nanofiber	−1.5 V *vs.* NHE	CO (65%)	8 h	27
Nanofiber				HCOOH (27%)		
Co_3_O_4_	0.1 M KHCO_3_	Ultrathin	−0.88 V *vs.* SCE	HCOOH (60%)	20 h	82
Ultrathin						
Co_3_O_4_/N-GO	0.1 M KHCO_3_	Nano cube	−0.95 V *vs.* SCE	HCOOH (83%)	8 h	83
Nano cube						
Crystal facet like Co_3_O_4_/rGO	0.5 M KHCO_3_	Crystal facet tailored	−0.4 V *vs.* RHE	C_2_H_5_OH (45.9%)	6 h	28
				H_2_C=CH_2_ (28.8%)		
Atomic Co layers	0.1 M Na_2_SO_4_	Nanosheet	−0.85 V *vs.* SCE	HCOOH (90%)	60 h	84
Co/CNT	0.5 M KHCO_3_	Polyhedral	−0.7 V *vs.* RHE	CO (90%)	40 h	85
Polyhedral						
Cu_2_O/rGO	0.1 M KHCO_3_	Cubic Cu_2_O anchored on rGO	−0.9 V *vs.* RHE	CO (70%)	1 h	86
P- and N-co-doped rGO	0.1 M KHCO_3_	Dual dopants	−0.9 V *vs.* RHE	HCOOH (70%)	—	87 and 88
Flower-like Co_3_O_4_	0.5 M NaHCO_3_	Flower	−0.62 V *vs.* Ag/AgCl	CH_3_COOH (69%)	2.5 h	This work
Flower-like Co_3_O_4_/P-rGO	0.5 M NaHCO_3_	Flower-like Co_3_O_4_	−0.62 V *vs.* Ag/AgCl	CH_3_COOH (58%)	2.5 h	This work
		Anchored on the P-rGO sheet		CH_3_CH_2_CHO (9%)		

## 4. Conclusion

We have synthesized flower-like Co_3_O_4_ and demonstrated its potential as an efficient catalyst for the electrochemical conversion of CO_2_ gas to ethanoic acid in aqueous bicarbonate medium, achieving a high faradaic efficiency of 69%. The catalytic activity was significantly enhanced by integrating flower-like Co_3_O_4_ with P-doped rGO, forming a hybrid catalyst that facilitated the selective production of ethanoic acid and propanal with 58% and 9% faradaic efficiencies, respectively. The hybrid system not only exhibits excellent product selectivity but also maintains robust electrochemical stability throughout the CO_2_ reduction process. The synergistic interaction between Co_3_O_4_ and P-rGO likely promotes multi-electron transfer pathways, enabling the formation of complex molecules such as propanal. This work provides mechanistic insight into CO_2_ electroreduction and opens a new avenue for producing renewable, value-added chemicals from greenhouse gas CO_2,_ and offers a promising strategy to address environmental and energy-related challenges.

## Author contributions

Rad Mosharrof Mim: writing – review & editing, writing – original draft, methodology, investigation, formal analysis. Md. Shamim-Alam – writing – review & editing, Sangjukta Yesmin: writing – review & editing. Md. Mominul Islam: writing – review & editing, investigation, formal analysis. Chanchal Kumar Roy: writing – original draft, methodology, formal analysis. Abu Bin Imran: writing – review & editing, writing – original draft, methodology, investigation, formal analysis, conceptualization. Al-Nakib Chowdhury: writing – review & editing, writing – original draft, project administration, methodology, investigation, funding acquisition, formal analysis, conceptualization.

## Conflicts of interest

There are no conflicts to declare.

## Supplementary Material

RA-016-D6RA01555G-s001

## Data Availability

All data supporting the findings of this study are included within the manuscript and its supplementary information (SI). No additional datasets were generated or analyzed. Supplementary information is available. See DOI: https://doi.org/10.1039/d6ra01555g.
