# Supplementary material for: Fabrication of Co3O4/P-rGO for electrocatalytic reduction of greenhouse CO2 gas into value-added chemicals in aqueous solution
Source: RSC Adv. 2026 Apr 13;16(21):19158–75. doi: 10.1039/d6ra01555g (PMC13071578; doi:10.1039/d6ra01555g)
Supplement: RA-016-D6RA01555G-s001 [file RA-016-D6RA01555G-s001.pdf]

Supplementary Information (SI) for RSC Advances.
This journal is © The Royal Society of Chemistry 2026

Fabrication of Co_3O_4/P-rGO for Electrocatalytic Reduction of Greenhouse CO_2 Gas into Value-added Chemicals in Aqueous Solution

Rad Mosharrof Mim[1], Md. Shamim Alam[2], Sangjukta Yesmin[1], Md. Mominul Islam[3], Chanchal Kumar Roy[1], Abu Bin Imran[1*], and Al-Nakib Chowdhury[1*]

[1]Department of Chemistry, Bangladesh University of Engineering and Technology, Dhaka-1000, Bangladesh

[2]Department of Textile Engineering, Southeast University, Tejgaon I/A, Dhaka, Bangladesh

[3]Department of Chemistry, University of Dhaka, Dhaka-1000, Bangladesh

Corresponding emails: abimran@chem.buet.ac.bd, nakib@chem.buet.ac.bd

1. Growth process of the Flower-like precursor

The growth dependence was examined to understand the growth mechanism of flower-like Co_3O_4. As shown in fig. S1(b), the flower-like structure forms within 180 minutes. In fact, the flower-like structure is composed of micro-sheets with an average length of 1.3 μm from the flower center, and the average diameter was 4 μm. After a fixed period at the same temperature and conditions (the same amount of solution, the same volume of autoclave), the 3D flower-like precursor gradually forms. Meanwhile, the precursor ratio had been adjusted for the specific morphological synthesis processes. In this synthesis, hexamethylenetetramine is used as a surface additive to promote morphological growth. Also, it plays an important role as a chelating agent. That's why the molar ratio of HMTA had been changed for the shape-controlled synthesis of Co_3O_4. When the molar ratio of HMTA was maintained at 0.5 mole, the flaky-type sheets were observed, as shown in Figure S1(a). Here, the flaky sheets become thinner, but curling is observed at the edge. However, when the molar ratio of HMTA is increased, as shown in Figure S1(b), the flower-like morphology was observed, with the sheets becoming thin and curling. This phenomenon is due to surface tension during the hydrothermal reaction, and ultrathin 2D nanostructures are more prone to crimping.

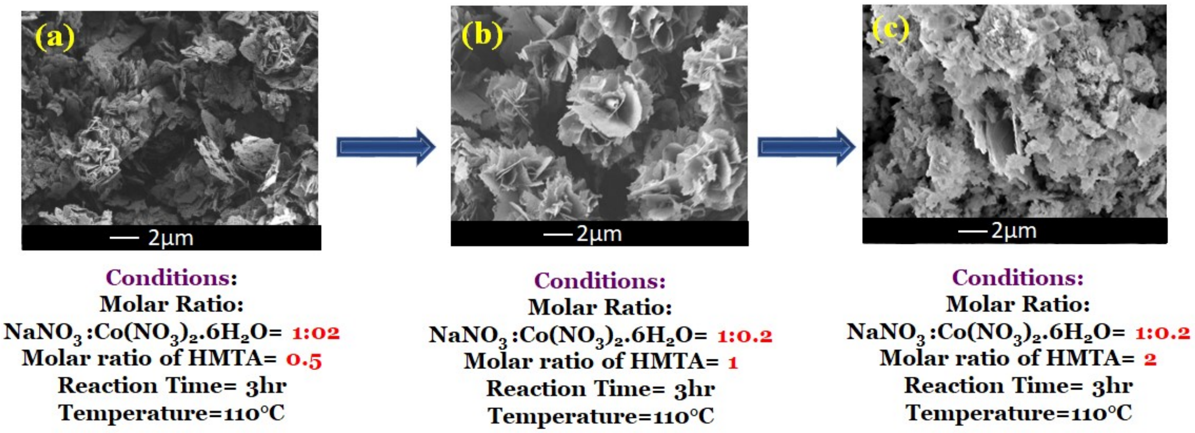

Figure S1. Growth mechanism of the flower-like Co_3O_4.

Figure S1(c) shows that the shape of the 3D flower structure is fully destroyed and the flaky-sheets are agglomerated, which is observed by the addition of HMTA in 4 times greater than the initial molar ratio of HMTA. Therefore, the formation process of the 3D flower-like precursor likely involves nucleation, followed by growth into a flower-like structure, continued development with nanosheet thinning and curling, and finally, the formation of the complete 3D flower-like samples. That's why the right number of precursor molar ratio is an important parameter for a shape-controlled synthesis of a sample. Reactions involving synthesis of flower-like Co_3O_4:

The reactions involved in hydrothermal synthesis can be illustrated as follows:

$$C_6H_{12}N_4 + 4H_2O \xrightarrow{Ethanol} 6HCHO + 4NH_3$$

$$NH_3 + H_2O \rightarrow NH_4^+ + OH^-$$

$$NaNO_3 + H_2O \rightarrow Na^+ + NO_3^-$$

$$Co(NO_3)_2.6H_2O + Na^+ \rightarrow Co^{2+} + NaNO_3$$

$$Co^{2+} + 2OH^- \rightarrow Co(OH)_2\downarrow (Green\ ppt.)$$

$$Co(OH)_2 \xrightarrow{\substack{Calcination \\ 400°C,\ 2h\ in \\ air\ atmoshpere}} Co_3O_4$$

2. XRD analysis of GO and P doped rGO:

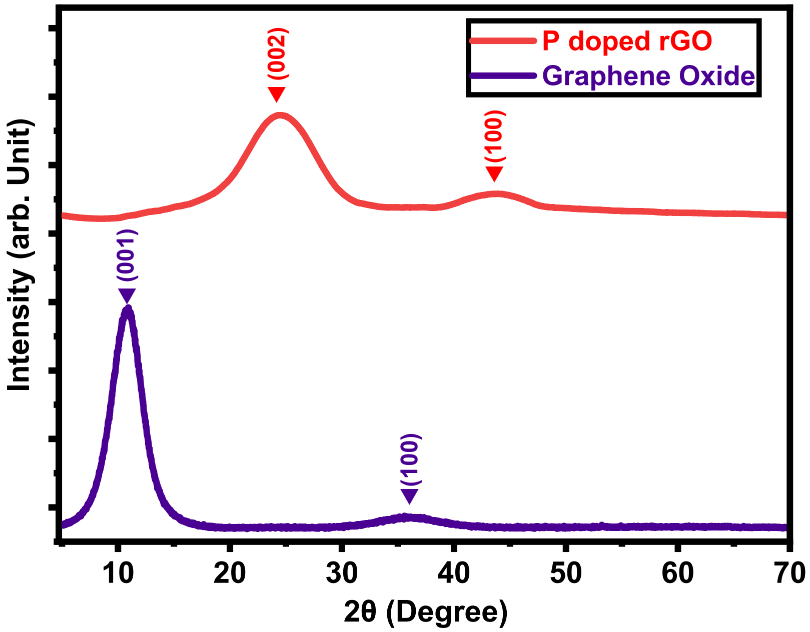

Figure S2. XRD pattern of Synthesized Graphene oxide and P-doped rGO

2. Energy-dispersive X-ray spectroscopy analysis

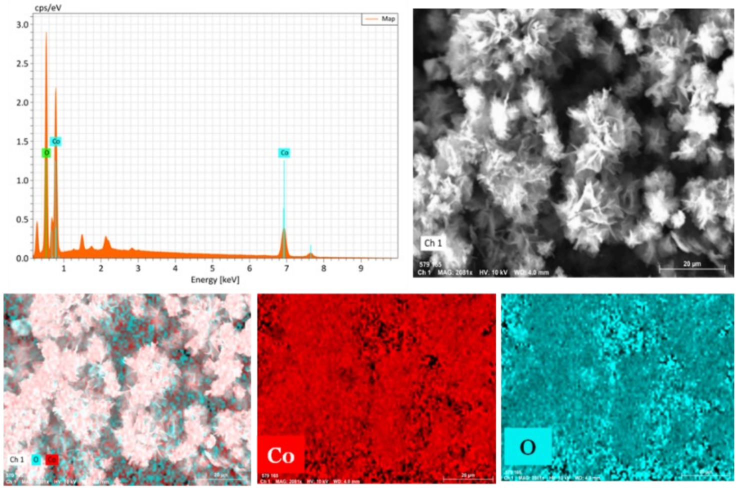

Figure S3. EDX analysis of (a) elemental spectrum of flower-like Co_3O_4 (b-c) EDX mapping of a selected area to identify Co and O elements; (d) Co element and (e) O element.

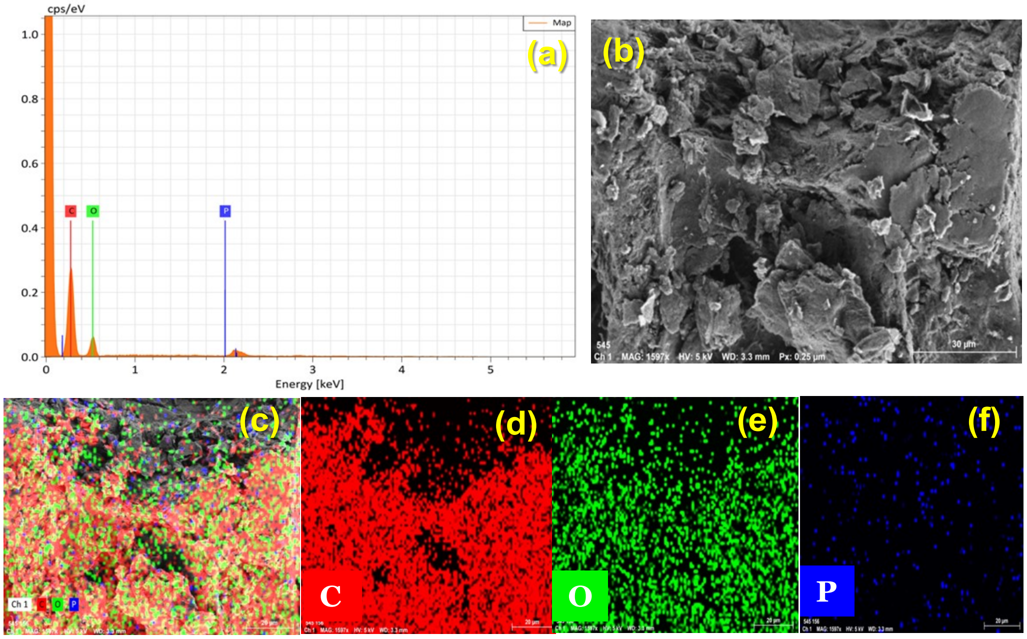

Figure S4. EDX of (a) elemental spectrum of P doped rGO, (b-c) EDX mapping of selected area of identifying C, O, and P elements, (d) C element, (e) O element, (f) P element.

3. Raman Spectra:

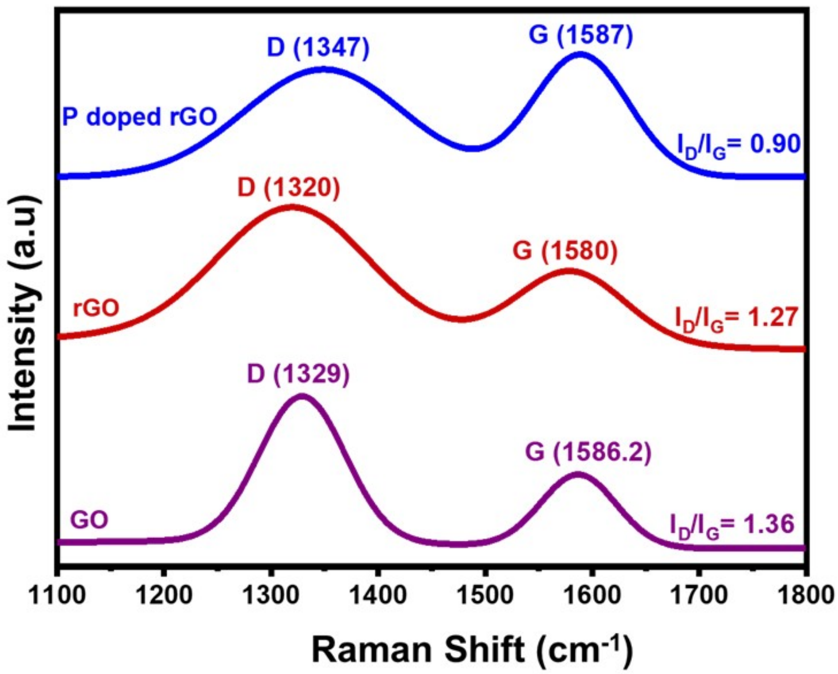

Figure S5. Raman Spectra of Synthesized GO, rGO and P doped rGO

3. Dopant Effects

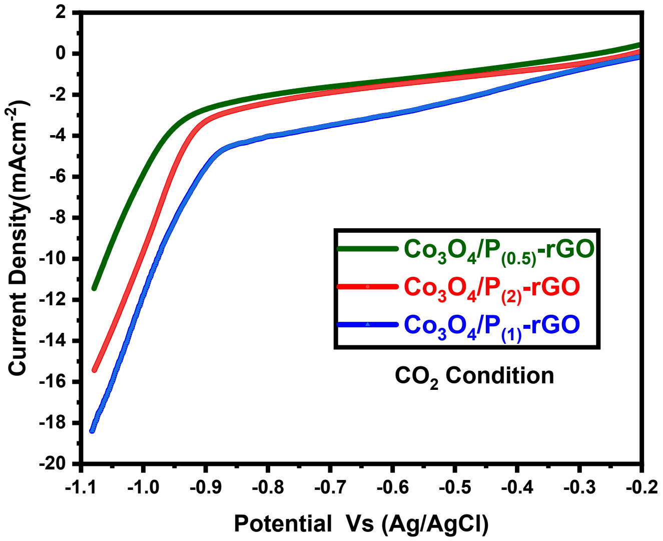

Figure S6. LSV for Modified electrode in CO_2 condition with different amount dopant composition including flower -like $Co_3O_4/P_{(0.5\%)}$-rGO (green), flower-like $Co_3O_4/P_{(2\%)}$-rGO (red) and flower-like $Co_3O_4/P_{(1\%)}$-rGO(blue) to hybrid materials in 0.5M $NaHCO_3$ electrolyte.

Table S1. LSV data for effect on dopant amount.

Amount of GO (g)	Amount of Dopant (g)	% of Dopant	Current Density ($mAcm^{-2}$)
0.2	1	0.5	-11.51
	2	1	-18.52

4. Tafel Plot analysis

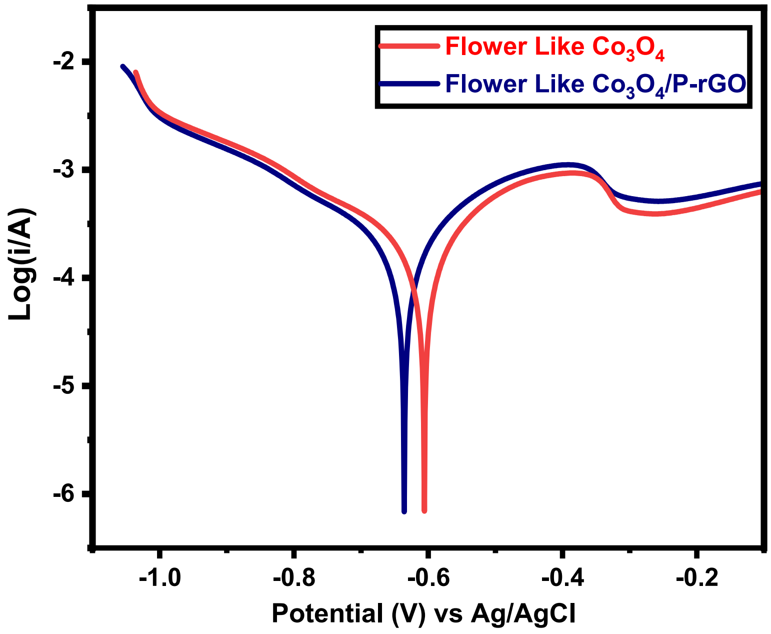

Figure S7. Tafel Plot Analysis for Modified electrode with flower-like Co_3O_4 (red) and flower-like $Co_3O_4/P_{(1\%)}$-rGO (blue) in $NaHCO_3$ electrolyte.

5. GC-MS Spectroscopy analysis:

The products of CO_2 reduction in a 0.5M $NaHCO_3$ electrolyte were analyzed using Gas Chromatography-Mass Spectroscopy (GC-MS). After completing the chronoamperometry process, the electrolyte samples were prepared and spiked with standard ethanol to facilitate quantification. Before injection, samples were filtered to remove particulates and diluted as necessary to fall within the linear range of the instrument. Quantification of the identified products was performed using calibration curves constructed from known concentrations of standard compounds, as shown in Figure S8.

The mass spectra of the detected analytes were compared with the NIST spectral library, confirming the presence of ethanoic acid and propanal, as summarized in the table. Retention times of the analytes were consistent with those of the corresponding standard compounds. In the flower-like Co_3O_4 catalyzed samples, ethanoic acid was the primary product detected, whereas in the hybrid Co_3O_4/P-rGO catalyzed samples, both ethanoic acid and propanal were identified. Each product was quantified by correlating the respective calibration curve, as shown in Figure 8(b).

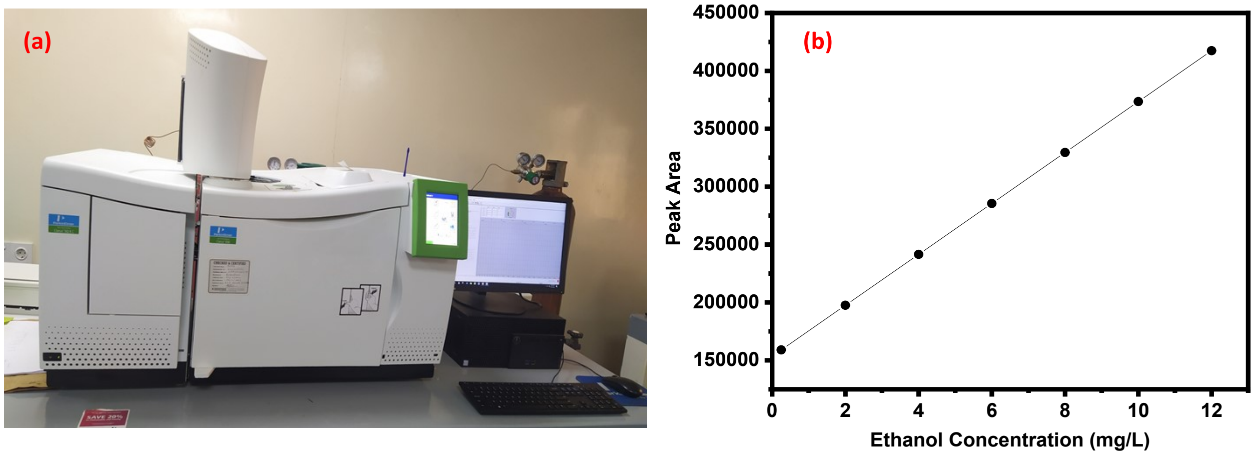

Figure S8. GC-MS analysis (a) GC-MS Spectrometer, (b) Calibration Curve of Known Standards

Table S2: GC–MS analysis of CO_2 reduction products in 0.5 M $NaHCO_3$ electrolyte using Co_3O_4 and Co_3O_4/P-rGO catalysts. Retention times, NIST mass spectral library identification numbers, chromatographic peak areas, and percentage peak areas detected products are summarized.

Catalyst	Product	Retention Time	NIST MS Number	Area	Total Area	%Area
Co_3O_4	Ethanoic Acid	3.47	227635	241524.05	539545165.9	0.4544
Co_3O_4 /P-rGO	Ethanoic Acid	3.40	227635	300466.893	373694818.5	0.0804
	Propanal	5.37	123386	177850.078	373694818.4	0.0459

6. Detailed calculation of faradaic efficiency:

After analyzing the electrolyte with GC-MS, the faradaic efficiency was evaluated with the equation (i) mentioned in the methodology. The equation,

$$\%FE = \frac{nFZ}{Q} \times 100 \qquad \text{............(i)}$$

Where, $n =$ the number of moles of the product (mole)

$Z =$ the number of electrons required to produce that given product

$F =$ Faraday constant

$Q =$ Total charge release (C)

For flower-like Co_3O_4,

The ethanoic acid was detected, and the amount was evaluated by correlating with a calibration curve of standard known compounds.

The mole of the product (ethanoic acid), $n_{ethanoic\ acid} = 1.6536\times10^{-6}$ mol

Faraday Constant, $F =$ 96500 C/mol

Total charge release for flower-like Co_3O_4 electrode, Q = 1.85 C

From the reaction mentioned in the mechanistic pathway (figure 13),

The number of electrons required for ethanoic acid formation, $Z_{ethanoic\ acid} = 8$

Then, faradaic efficiency,

$$\%FE_{ethanoic\ acid} = \frac{n_{ethanoic\ acid} \times 96500 \times Z_{ethanoic\ acid}}{Q} \times 100$$

$$= \frac{1.653 \times 10^{-6} \times 96500 \times 8 \times 100}{1.85} = 69\%$$

For flower-like Co_3O_4/P-rGO composite,

Ethanoic acid and propanal were detected, and both concentrations were evaluated by correlating them with a calibration curve of known standard compounds.

For ethanoic acid,

The mole of the product (ethanoic acid), $n_{ethanoic\ acid}$ = 2.752×10^{-6} mol

Faraday Constant, $F =$ 96500 C/mol

Total charge release for flower-like Co_3O_4 /P-rGO electrode, Q = 3.65 C

From the reaction mentioned in the mechanistic pathway (Figure 13),

The number of electrons required for ethanoic acid formation, $Z_{ethanoic\ acid}$ = 8

Then, the faradaic efficiency,

$$\%FE_{ethanoic\ acid} = \frac{n_{ethanoic\ acid} \times 96500 \times Z_{ethanoic\ acid}}{Q} \times 100$$

$$= \frac{2.752 \times 10^{-6} \times 96500 \times 8 \times 100}{3.65} = 58\%$$

For Propanal,

The mole of the product (Propanal), $n_{Propanal}$ = 2.128×10^{-7} mol

Faraday Constant, $F =$ 96500 C/mol

Total charge release for flower-like Co_3O_4 electrode, Q = 3.65 C

From the reaction mentioned in the mechanistic pathway (Figure 13),

The number of electrons required for ethanoic acid formation, $Z_{ethanoic\ acid} = 16$

Then, faradaic efficiency,

$$\%FE_{ethanoic\ acid} = \frac{n_{propanal} \times 96500 \times Z_{propanal}}{Q} \times 100$$

$$= \frac{2.752 \times 10^{-7} \times 96500 \times 16 \times 100}{3.65} = 9\%$$
